# Evocative effects on the early caregiving environment of genetic factors underlying the development of intellectual and academic ability

**DOI:** 10.1111/cdev.14142

**Published:** 2024-07-30

**Authors:** Chloe Austerberry, Pasco Fearon, Angelica Ronald, Leslie D. Leve, Jody M. Ganiban, Misaki N. Natsuaki, Daniel S. Shaw, Jenae M. Neiderhiser, David Reiss

**Affiliations:** ^1^ Centre for Family Research University of Cambridge Cambridge UK; ^2^ Research Department of Clinical, Educational and Health Psychology UCL London UK; ^3^ School of Psychology University of Surrey Guildford UK; ^4^ Prevention Science Institute University of Oregon Eugene Oregon USA; ^5^ Cambridge Public Health University of Cambridge Cambridge United Kingdom of Great Britain and Northern Ireland; ^6^ Department of Psychology George Washington University Washington District of Columbia USA; ^7^ Department of Psychology University of California Riverside California USA; ^8^ Department of Psychology University of Pittsburgh Pittsburgh Pennsylvania USA; ^9^ Department of Psychology The Pennsylvania State University University Park Pennsylvania USA; ^10^ Yale Child Study Center Yale School of Medicine New Haven Connecticut USA

## Abstract

This study examined gene–environment correlation (*r*GE) in intellectual and academic development in 561 U.S.‐based adoptees (57% male; 56% non‐Latinx White, 19% multiracial, 13% Black or African American, 11% Latinx) and their birth and adoptive parents between 2003 and 2017. Birth mother intellectual and academic performance predicted adoptive mother warmth at child age 6 (*β* = .14, *p* = .038) and 7 (*β* = .12, *p* = .040) but not 4.5 years, and adoptive father warmth at 7 (*β* = .18, *p* = .007) but not 4.5 or 6 years. These *r*GE effects were not mediated by children's language. Contrary to theory that *r*GE accounts for increasing heritability of intellectual ability, parenting did not mediate genetic effects on children's language or academic performance.

**Abbreviations**
CHAOS: Chaos, Hubbub, and Order Scale
CLPM: cross‐lagged panel model
DIBELS: Dynamic Indicators of Basic Early Literacy Skills
EduYears: years of education
EFA: exploratory factor analysis
EGDS: Early Growth and Development Study
E‐Risk: Environmental Risk
FIML: full information maximum likelihood
HLE: Home Literacy Environment
IOWA: Iowa Family Interaction Rating Scales
ISF: Initial Sound Fluency
LNF: Letter Naming Fluency
MAR: missing at random
MCAR: missing completely at random
NWF: Nonsense Word Fluency
*r*GE: gene–environment correlation
RI: random‐intercept
RI‐CLPM: random intercept cross‐lagged panel model
SEM: structural equation modeling
SES: socioeconomic status
TOPEL: Test of Preschool Early Literacy

Intellectual and academic performance are powerful predictors of health and success across the lifespan (Deary et al., 2010; Hummer & Hernandez, 2013; Kosik et al., 2018). They are both moderately to highly heritable (Bouchard & McGue, 1981; de Zeeuw et al., 2015; Haworth et al., 2010; Kovas et al., 2013), and there is high genetic overlap between them (Davis et al., 2009; Plomin & Kovas, 2005). Although the literature is somewhat mixed as to whether the heritability of intellectual and academic performance increases, decreases or remains stable across childhood, in part because of wide and overlapping heritability estimates at different age ranges (e.g., Kovas et al., 2013), overall, there appears to be a general increase in the heritability of academic performance from early to middle childhood (Andreola et al., 2021; Austerberry, Mateen, et al., 2022; de Zeeuw et al., 2015; Verhoef et al., 2021). For example, meta‐analysis of twin studies indicates that language is approximately 24% heritable in infancy (Austerberry, Mateen, et al., 2022), whereas from middle childhood onwards, pooled heritability estimates from meta‐analyses of language, literacy and other academic skills range from 34% to 80% (Andreola et al., 2021; de Zeeuw et al., 2015). There is also strong and replicable evidence of a linear increase in the heritability of general intellectual ability across the lifespan (Bouchard & McGue, 1981; Haworth et al., 2010). Based on the stability of the genome, this increasing heritability seems somewhat paradoxical. However, a plausible explanation for increasing heritability—which we aimed to examine in early intellectual and academic development—is that genetic differences are amplified across time as individuals influence, select and evoke differences in their environments that are correlated with their genotype (Plomin et al., 1977; Scarr & McCartney, 1983). Three main forms of gene–environment correlation (*r*GE) have been defined in the literature (Plomin et al., 1977; Scarr & McCartney, 1983): active, evocative (also known as reactive), and passive *r*GE—for definitions and examples of each, see Table 1.

**TABLE 1:** Definitions and examples of *r*GE.

Type of *r*GE	Definition	Example
Evocative	Occurs when an individual's genetically influenced behaviors evoke or elicit specific responses from their environment	A child with a genetic propensity toward high academic achievement may display traits and behaviors that evoke more cognitive stimulation from their parents (e.g., reading to the child and helping them with homework more frequently), thereby creating an environment that is conducive to learning
Active	Occurs when an individual's genetically influenced traits or behaviors lead them to actively select or seek out certain environments	A child with a genetic propensity toward high academic achievement might choose to read more or participate in extracurricular activities, thereby placing themselves in environments that enhance their academic skills
Passive	Occurs when a parent's genetically influenced traits or behaviors shape the environment their child is exposed to. Because the parent and child share genes, this environment (which is correlated with the parent's genes) will be correlated with the child's genes. Unlike the other two forms of *r*GE, the correlation between the child's genes and their environment does not reflect a causal link directly between the child's genes and the environment they are exposed to, but rather between the parents' genes and the environment they create for the child	A parent with a genetic propensity toward high academic achievement may create an academically enriched environment at home. Although the child's genetic propensity toward academic achievement is correlated with this environment, their exposure to it is not due to their own genetic predispositions or actions but rather those of their parent

Abbreviations: *r*GE, gene–environment correlation.

As young children have limited opportunities to select their environments, the form of *r*GE that may be particularly relevant during early childhood is evocative *r*GE, which occurs when an individual's genetically influenced characteristics systematically evoke differences in their environment (Plomin et al., 1977; Scarr & McCartney, 1983). In the context of intellectual and academic development, children's education‐associated genetic differences may underlie early behavioral differences (e.g., vocabulary and interest in toys and books) that systematically elicit differences in the caregiving environment (e.g., warm and responsive parenting) and mediate genetic effects on academic outcomes, potentially amplifying initial genetic differences via environmental mechanisms. Furthermore, although active selection of the environment may be more limited in childhood than in adolescence and adulthood, to a certain extent young children's genetic propensities may also lead them to actively select environmental exposures (active *r*GE) that may be advantageous or detrimental for intellectual and academic development (e.g., seeking out books to read or showing interest in television or video games). As these evoked or selected environmental mechanisms would be correlated with genetic differences, they could plausibly be masked by heritability estimates. This hypothesis is described in depth by Dickens and Flynn (2001), who explore the possibility that this amplification process accounts for rising levels of intelligence across the lifespan (and in successive cohorts of children and adults). They suggest that initially small genetic differences may become more potent over time through a multiplier effect produced by mechanisms of reciprocal causation between individuals' genotypes and the environmental influences they seek out or evoke. This explanation is challenging to test because it requires a genetically sensitive design and extensive longitudinal data. Consequently, to our knowledge, the Dickens and Flynn (2001) hypothesis has never been empirically tested in intellectual and academic development. However, there is good reason to believe that children's early characteristics can elicit responses in the caregiving environment, evidenced by a robust body of literature supporting the existence of interplay between child traits and the home and parenting environment. This includes decades of evidence from phenotypic research of bidirectional effects between parents and children (Hipwell et al., 2008; Lugo‐Gil & Tamis‐LeMonda, 2008; Pardini et al., 2008) and the potential influence of child characteristics on parenting behavior (Bell, 1968). Furthermore, behavioral genetics research provides evidence of possible evocative and active influences of children's genes on parenting and the home environment. For example, there is a substantial literature from twin and parent‐offspring adoption studies (e.g., the Twins Early Development Study, Quebec Newborn Twin Study, and Early Growth and Development Study [EGDS]) demonstrating possible evocative effects of children's genes on differences in parenting, including in early childhood (Boivin et al., 2005; Cheung et al., 2023; Elam et al., 2014; Fearon et al., 2015; Harold et al., 2013; Knafo & Plomin, 2006; Plomin & Bergeman, 1991). However, only a small subset of this literature (outlined below) is specifically focused on evocative or active effects of genetic influences underlying intellectual and academic development.

Some phenotypic evidence suggests that children's intellectual differences may evoke differences in parenting. For example, there is evidence of longitudinal bidirectional associations between children's intellectual performance and parental responsiveness, cognitive stimulation and sensitivity between 14 months and 5 years of age from two studies of children and their parents—the Family Life Project (*N* = 1292) and the Early Head Start Research and Evaluation Study (*N* = 2089) (Blair et al., 2014; Lugo‐Gil & Tamis‐LeMonda, 2008). Furthermore, some genetically informative literature demonstrates that child → parent (or child → household environment) effects in intellectual development may be partly genetically driven. For example, using longitudinal data from a sample of 650 monozygotic and dizygotic twins, Tucker‐Drob and Harden (2012) found that, after controlling for parental cognitive stimulation at age 2 years, children's intellectual performance (a composite of verbal and nonverbal cognitive ability) at age 2 predicted cognitively stimulating parenting at age 4 years. This association was predominantly mediated by genetic variation, indicating that genetically influenced differences in the child may evoke responses from their caregivers. Additionally, using data from 860 mothers and children in the Environmental Risk (E‐Risk) Study, Wertz et al. (2020) found that children's polygenic scores for years of education (EduYears) predicted maternal positive parenting (cognitive stimulation, warmth and sensitivity), and home chaos when children were between 5 and 10 years of age, after controlling for the direct effect of mothers' EduYears polygenic scores. There is also evidence from unrelated individuals in the Twins Early Development Study that children's education polygenic scores were associated with aspects of their early caregiving, such as breastfeeding duration, whether the TV is usually on in the home, parental smacking, and number of books in the home (Krapohl et al., 2017). However, as the study did not control for parental genetics, it was not possible to rule out the likelihood that passive *r*GE (defined in Table 1) partly contributed to these associations.

The adoption design is ideally suited to investigate evocative and active *r*GE as, unlike all previous studies of evocative or active *r*GE in cognitive and academic development, it eliminates passive *r*GE while also being able to test the role of genetically mediated traits in evoking or selecting different responses within the family home. However, evocative and active *r*GE in intellectual and academic development have never been examined using an adoption design. The present analyses aimed to address this gap in the literature by using a prospective parent–offspring adoption study to examine whether genetic influences that contribute to the development of children's intellectual ability lead children to evoke or actively select differences in their early caregiving environment.

We also aimed to test the Dickens and Flynn (2001) hypothesis by examining whether any observed differences in the caregiving environment would mediate genetic effects on later academic performance. To our knowledge, no previous research has tested whether observed evocative or active effects on the caregiving environment predict subsequent intellectual or academic outcomes. However, developmental research has uncovered several features of the rearing environment in childhood and adolescence that predict subsequent intellectual and academic development in childhood and adolescence. One of the most well‐established constructs is positive parenting, which includes parental involvement, proactive anticipation of children's needs, responsivity, and warmth (Fan & Chen, 2001; Lugo‐Gil & Tamis‐LeMonda, 2008; Madigan et al., 2019). For example, in a meta‐analysis of 37 cross‐sectional and longitudinal studies of parenting and early childhood language (*M* = 33.5 months; range = 12–71 months), the pooled effect sizes were positive for the association between sensitive‐responsive parenting and child language (*r*_pooled_ = .27) and warm parenting and child language (*r*_pooled_ = .16) (Madigan et al., 2019). Additionally, in a meta‐analysis of 92 cross‐sectional and longitudinal studies of parenting and academic performance in childhood and adolescence, there was a positive association (*r*_pooled_ = .25) between parental involvement and academic performance (Fan & Chen, 2001). Other potentially important mechanisms include opportunities for learning and reading (Taylor et al., 2004) and low levels of family chaos (Johnson et al., 2008; Petrill et al., 2004). For example, in a sample of *n* = 2337 twins from the Twins Early Development Study, family chaos and children's academic achievement were significantly negatively associated (*r* = −.26), and this association appeared to be partly (37%) explained by genetic factors (Hanscombe et al., 2011). Use of screen media in the home, including television, the internet and video games, also appears to be negatively associated with intellectual and academic performance (Ribner et al., 2017; Shin, 2004). It is worth noting that the literature on screen use is somewhat mixed compared to the literature on the promotive effects on intellectual and academic development of positive and cognitively stimulating parenting. Two large meta‐analyses found that screen time in general tends to be negatively associated (*r* = −.29 and *r* = −.14, respectively) with language and academic performance in childhood and adolescence (Adelantado‐Renau et al., 2019; Madigan et al., 2020). However, one of these meta‐analyses also found that early childhood language was positively associated with higher‐quality (*r*_pooled_ = .13) and parent‐monitored screen time (*r*_pooled_ = .16) (Madigan et al., 2020). Based on this literature, we chose to focus our analyses primarily on positive parenting and secondarily on less widely researched or more mixed dimensions of the caregiving environment, such as household chaos and screen time.

Finally, we aimed to examine which genetically influenced child characteristics might lead children to evoke or select differences in the caregiving environment. In prior research, children's language performance at 4.5 and 6 years of age appeared to be an early manifestation of genetic influences on intellectual and academic abilities at age 7 years and potentially also in adulthood (as adoptee language at 4.5 and 6 years was associated with their adult birth parent's intellectual and academic performance) (Austerberry, Fearon, et al., 2022). This research identifying early language as a likely mediator of genetic influences on later intellectual outcomes informed our decision to examine whether children's language‐mediated associations between genetic influences on children and their caregiving environment.

We addressed our aims by testing the following three hypotheses in longitudinal cross‐lagged panel models (CLPM) with the paths displayed in Figure 1. First, we tested the hypothesis that genetic influences contributing to children's intellectual and academic performance would have evocative effects on the parenting warmth they received in early childhood and, in exploratory analyses, evocative or active effects on the levels of household chaos and household and child screen media use. Specifically, we expected that birth parent intellectual and academic performance (used as a proxy for genetic influences on children's intellectual and academic performance) would predict parenting warmth when children were 4.5, 6, and 7 years of age (and in the Supporting Information, negatively predict household chaos and screen media use). Second, we tested the Dicken's and Flynn theory by examining the hypothesis that evocative effects on parenting warmth (and evocative or active effects on home chaos and screen media use) would mediate genetic effects on intellectual and academic performance. Specifically, we expected that adoptive parent warmth (and exploratorily, household chaos and screen media use) when children were 4.5 or 6 years of age, or both, would mediate positive associations between birth parent intellectual performance and children's language at 6 or 7 years of age, or both. Third, we tested the hypothesis that children's early language performance at 4.5 or 6 years, or both, would mediate the associations between birth parent intellectual performance and adoptive parent warmth (and household chaos and screen media use) when children were 6 or 7 years of age, or both. Although this is the first research to test Hypotheses 2 and 3, and the first to test hypothesis 1 using an adoption design, on a continuum from exploratory to confirmatory, our hypotheses are largely confirmatory because they are directional and grounded in converging bodies of robust literature.

**FIGURE 1:**
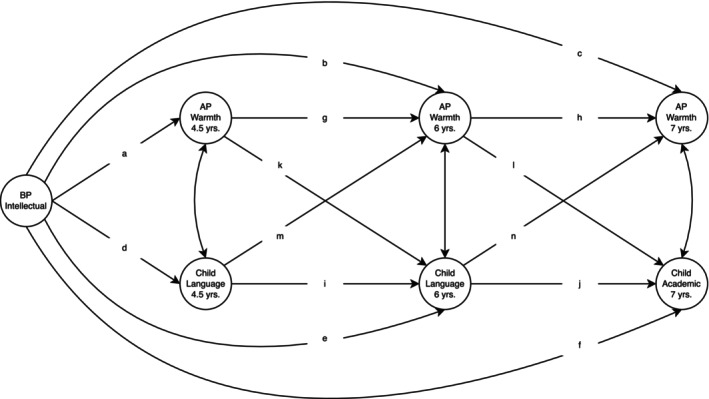
Paths in cross‐lagged panel model. Hypothesis 1 will be tested by examining the direct effects of birth parent intellectual performance on adoptive parent warmth (paths *a*, *b*, and *c*), the indirect effects of birth parent intellectual performance on adoptive parent warmth (paths *ag*, *agh*, and *bh*) and the total effects of birth parent intellectual performance on adoptive parent warmth (paths *b* + *ag* + *dm*, and *c* + *agh* + *akn* + *bh* + *dmh* + *din* + *en*). Hypothesis 2 will be tested by examining the indirect effects of birth parent intellectual performance on child language and academic performance via parental warmth (paths *ak*, *akj*, *bl*, *agl*, and (*b* + *ag* + *dm*)*l*). Hypothesis 3 will be tested by examining the indirect effects of birth parent intellectual performance on parental warmth via child language (*dm*, *dmh*, *en*, *din*, and (*e* + *di* + *ak*)*n*). AP, adoptive parent; BP, birth parent.

## METHOD

### Participants

Participants were 561 linked sets of adopted children and their birth mothers (*n* = 554), birth fathers (*n* = 210), and adoptive parents (562 adoptive fathers and 569 adoptive mothers) from the EGDS, a U.S.‐based, longitudinal, prospective parent–child adoption study (Leve et al., 2013, 2019). The numbers of adoptive mothers and fathers do not sum to 561 because the sample includes 41 same‐sex parent families and 15 additional adoptive parents who entered the family after the original couple adopted the child.

Participants were recruited through 45 adoption agencies in 15 states across the United States (Leve et al., 2019) in two cohorts: First, in 2003–2013, a sample of 361 adopted children and their birth and adoptive families and, second, in 2007–2017, 200 children and their families. EGDS assessments occurred in intervals of 9 months to 2 years and are still ongoing. We used data collected from birth parents at 9 months, 18 months, and 4.5 years postpartum and from adoptive parents and adoptees when adoptees were 9, 18, and 27 months of age, and 4.5, 6 and 7 years of age. At each of these time points, two adoptive parents were invited to participate. As Adoptive Parent 1 was most often the adoptive mother (96.6%) and Adoptive Parent 2 was most often the adoptive father (95.8%), Adoptive Parent 1 will be referred to from now on as adoptive mother and Adoptive Parent 2 as adoptive father.

The adopted children in the sample were 57% male. Adoptees were 56% non‐Latinx White, 19% multiracial, 13% Black or African American, 11% Hispanic or Latinx and <1% “other” (including Asian, American Indian, and unknown ethnicity). The mean age at which the children were placed for adoption was 5.58 days postpartum (SD = 12.4; median = 2; range = 0–91). Adoptive parents were predominantly non‐Latinx White (adoptive mothers: 92%; adoptive fathers: 90%). The remainder were Black or African American (adoptive mothers: 4%; adoptive fathers: 5%), Hispanic or Latinx (adoptive mothers: 2%; adoptive fathers: 2%), multiracial (adoptive mothers: 1%; adoptive fathers: 1%), and “other” (adoptive mothers: 1%; adoptive fathers: 2%). When the adoption took place, adoptive parents tended to be in their late thirties (adoptive mothers: *M* = 37.4 years, SD = 5.6; adoptive fathers: *M* = 38.3 years, SD = 5.8), married or cohabiting (adoptive mothers: 98%, adoptive fathers: 100%), college educated (adoptive mothers: 87%; adoptive fathers: 81%), and with a combined median income above $100,000. Most of the birth parents were non‐Latinx White (birth mothers: 70%; birth fathers: 70%). The remainder were Black or African American (birth mothers: 13%; birth fathers: 12%), Hispanic or Latinx (birth mothers: 7%; birth fathers: 10%), multiracial (birth mothers: 5%; birth fathers: 5%), and “other” (birth mothers: 5%; birth fathers: 4%). When the adoption took place, birth parents had a median household income of below $25,000, tended to be in their mid‐twenties (birth mothers: *M* = 24.4 years, SD = 6.0; birth fathers: *M* = 26.1 years, SD = 7.8), not married or cohabiting (birth mothers: 6.1%; birth fathers: 14.0%), and not college educated (birth mothers: 75%; birth fathers: 84%). There is no evidence of selective placement in EGDS (Leve et al., 2019). Additional information about the recruitment, composition and representativeness of the study is reported elsewhere (Leve et al., 2019).

### Ethics

Ethical approval was obtained from institutional review boards at the University of Oregon (Protocol number: 0304201400) and the Pennsylvania State University (Submission ID: CR00007591). Informed consent was obtained from all adult participants before research participation, and assent was obtained from children beginning at age 7 years.

### Measures

#### Birth parent general intellectual and academic performance

As a proxy for genetic influences on adopted children's intellectual and academic development, we created the same five‐indicator latent variables of birth mother and birth father general intellectual and academic performance used in Austerberry, Fearon, et al. (2022). The rationale for combining these items was based on the generalist genes literature, which demonstrates high genetic overlap between intellectual and academic abilities and disabilities (Davis et al., 2009; Plomin & Kovas, 2005), as well as the internal consistency (birth mother *α*_R_ = .84; birth father *α*_R_ = .85) and bivariate correlations among measures of birth parent intellectual and academic performance in the EGDS sample (range: *r* = .35–.70, full results reported in Austerberry, Mateen, et al., 2022). The first item was a standardized total score on the 28‐item Information subtest of the Wechsler Adult Intelligence Scale (Wechsler, 1997), administered to birth parents at 18 months postpartum. It is considered to be a representative measure of *g* (*g* loading = .79). The remaining four indicators were *T*‐scores from subtests of the Woodcock–Johnson Tests of Achievement III (Woodcock et al., 2001), administered to birth parents at 4.5 years postpartum: (1) 76‐item Letter‐Word Identification, measuring reading decoding; (2) 32‐item Word Attack, capturing decoding and phonetic coding; (3) 98‐item Reading Fluency, measuring reading speed and semantic processing speed; (4) 160‐item Math Fluency, indexing math, and numerical performance. Previous research suggests that the reading and math subscales are well correlated with other established measures of intellectual ability and have split‐half reliability and internal consistency of above .80 (often above .90) (Timothy & Donald, 2003; Woodcock et al., 2001). The internal consistency of the five indicators in the EGDS sample was good (birth mother *α* = .84; birth father *α* = .85).

#### Child language

We created the same latent variables measuring language at 4.5 and 6 years of age as were used in Austerberry, Fearon, et al. (2022). The latent variable at age 4.5 years had three indicators, each of which was a subscale from the Test of Preschool Early Literacy (TOPEL) (Lonigan et al., 2011): (1) 36‐item Print Knowledge, measuring knowledge of the alphabet, written language conventions and written form; (2) 35‐item Definitional Vocabulary, assessing definitional and single‐word oral vocabulary; (3) 27‐item Phonological Awareness, measuring word elision and blending. Standard scores were used, derived from the distribution of the raw scores. Previous research suggests that the TOPEL has high internal consistency (*α* = .86–.96) and test–retest reliability (*r* = .81–.89), moderate predictive validity (*r* = .40–.62), and moderate to high concurrent validity (*r* = .59–.77) (Lonigan et al., 2011). In the EGDS sample the internal consistency of the three indicators was acceptable (*α* = .62). The latent variable at age 6 years had five indicators, one of which was a standardized score from the vocabulary assessment of the Wechsler Preschool and Primary Scale of Intelligence III (Wechsler, 2002), and four of which were assessments from the Dynamic Indicators of Basic Early Literacy Skills (DIBELS) (Good & Kaminski, 2002): (1) 16‐item Initial Sound Fluency (ISF), measuring phonemic awareness; (2) Letter Naming Fluency (LNF), capturing proficiency in naming upper‐ and lower‐case letters, using a list of 110 letters; (3) Phoneme Segmentation Fluency, assessing proficiency in fluently segmenting three‐ and four‐phoneme words into their individual phonemes, using a list of 24 words; (4) Nonsense Word Fluency (NWF), testing understanding of the alphabetic principle, including letter‐sound correspondence, using a list of 50 nonsense words. Previous research has found that ISF and LNF have good test–retest reliability (*r* = .88–.93) and robustly predict later reading performance (Kaminski & Good, 1996). Raw scores (representing the number of correct answers in 1 min) were converted to percentiles, reflecting performance relative to same grade‐level peers, based on nationally standardized normed scores (Good & Kaminski, 2002). The internal consistency of the five indicators in the EGDS sample was good (*α* = .76). Additional information on the rationale for combining these items is reported in Austerberry, Fearon, et al. (2022).

#### Child academic test performance

We created the same latent variable to estimate child academic test performance at age 7 years as Austerberry, Fearon, et al. (2022), drawing on four indicators of reading and math performance that were administered to birth parents from the Woodcock–Johnson Tests of Achievement III (Woodcock et al., 2001). The internal consistency of the four indicators in the EGDS sample was good (*α* = .88). Additional information on the rationale for combining these items is reported in Austerberry, Fearon, et al. (2022).

#### Caregiving environment

Prior to hypothesis testing, we conducted a split‐half exploratory factor analysis (EFA) of items from questionnaires on positive parenting, the home learning environment, and household chaos, administered to adoptive parents when the children were 4.5, 6, and 7 years of age. Results from the EFA are reported in the Supporting Information. Based on the factor structure of the items, we constructed the following three latent variables in our main analyses (as well as latent variables on chaos and screen use in exploratory analyses reported in the Supporting Information):

##### Parenting warmth

For use in our main analyses, we created a latent variable measuring parenting warmth at 4.5, 6, and 7 years of age using the six self‐report indicators from the Warmth subscale of the Iowa Family Interaction Rating Scales (IOWA) (Melby & Conger, 2001), administered to adoptive mothers and fathers. Each item was scored on a 7‐point scale from 1 (Never) to 7 (Always). The psychometric information for the IOWA is reported by Melby and Conger (2001). The internal consistency of the items in our sample was good (*α* = .87, .86, .88, for adoptive mother ratings at 4.5, 6, and 7 years, respectively; *α* = .85, .87, and .87, for adoptive father ratings).

##### Household chaos

For use in the exploratory analyses reported in the Supporting Information, we constructed a latent variable measuring household chaos when children were aged 4.5, 6, and 7 years of age using three indicators from the Chaos, Hubbub, and Order Scale (CHAOS) (Matheny et al., 1995): (1) “You can't hear yourself think in our home,” (2) “It's a real zoo in our home,” and (3) “The atmosphere in our house is calm” (reverse scored). Each item was rated on a 5‐point scale from 1 (Definitely Untrue) to 5 (Definitely True). The psychometric information for the CHAOS is provided by Matheny et al. (1995). Adoptive mother and adoptive father responses to each of the three items were correlated at all time points (*r* range: .31–.52, *p* < .001). Consequently, when data were available from both adoptive parents a mean score was used. The internal consistency of the three adoptive parent composite items in the sample was good at each timepoint (*α* = .84, .86, and .86 at 4.5, 6, and 7 years, respectively).

##### Screen media use

For use in the exploratory analyses reported in the Supporting Information, we created a latent variable measuring household and child screen media use when children were aged 4.5, 6, and 7 years of age using four adoptive parent‐rated indicators. Three items were from the Home Literacy Environment (HLE) questionnaire (Niklas & Schneider, 2013): “On average, how many hours per day does your child watch television or play video games?” on (1) “Weekdays,” (2) “Saturday,” (3) “Sunday.” One indicator was from the CHAOS Matheny et al. (1995): “There is usually a television turned on somewhere in our home,” rated on a 5‐point scale from 1 (Definitely Untrue) to 5 (definitely true). The psychometric information for the HLE is reported by Niklas and Schneider (2013) and the psychometric information for the CHAOS is reported by Matheny et al. (1995). Adoptive mother and adoptive father responses to each of the three items were correlated at all timepoints (*r* range: .39–.73, *p* < .001). Consequently, when data were available from both adoptive parents, a mean score was used. The internal consistency of the four adoptive parent composite items in the sample was good at each timepoint (*α* = .86, .83, .87 at 4.5, 6 and 7 years of age, respectively).

#### Covariates

Adoption openness, child sex, and prenatal risk were included as covariates. Adoption openness, which reflects ongoing contact between birth parents, adoptive parents, and the adoptee, was measured using a 4‐item measure (Ge et al., 2008) averaged across ratings provided at 9, 18, and 27 months postpartum by birth mothers and adoptive parents. Openness is important to control for in adoption studies because ongoing contact between birth parents and adoptees may increase similarities between birth parents and adoptees via environmental mechanisms, resulting in overestimates of heritability. As well as controlling for adoption openness by including it as a covariate in the analyses, we also conducted a sensitivity analysis that included the interaction term (birth parent intellectual ability × openness), testing whether birth parent effects were the same in open adoptions as in relatively closed ones. Prenatal risk (e.g., neonatal complications, prenatal drug use, prenatal exposure to toxins) was measured using a weighted total score derived from birth mother reports at 5 months postpartum and medical record data. Prenatal risk is important to control for in adoption studies because it can result in overestimated heritability if it leads to similarities between birth mother and adoptee due to the prenatal environment.

### Data analysis

The analyses were preregistered with the Open Science Framework in March 2021 (https://osf.io/pz67m). We conducted longitudinal CLPM in the lavaan package (Rosseel, 2012) in R 4.1.2, using structural equation modeling (SEM). SEM combines a measurement model, also known as confirmatory factor analysis, with a structural model testing the proposed causal relations. The full CLPM (displayed in Figure 1) was built in several steps, described in the Supporting Information. In recent years, the CLPM has received criticism for failing to distinguish within‐person processes from stable between‐person differences, and it has been suggested that this issue can be addressed by including a random intercept (Hamaker et al., 2015). Although the RI‐CLPM is more suitable for testing some hypotheses, the traditional CLPM was better able to test the hypotheses of the present study than the random‐intercept model (RI‐CLPM) for several reasons. First, we were examining the mediated effects of a stable, time‐invariant, and trait‐like, predictor (the genetic influences) on the cross‐lagged outcomes (parenting and child language and academic test performance). However, the variations of the RI‐CLPM that incorporate time‐invariant predictors, either predicting the observed variables or random intercepts, are not able to examine the mediated effects of a time‐invariant predictor on the cross‐lagged outcomes because they isolate the within‐person process from the between‐person process (Mulder & Hamaker, 2021; Rohrer & Murayama, 2023). Second, we were interested in examining the cascading transfer of effects of the stable, time‐invariant, genetic predictor, via the auto‐regressive and cross‐lagged paths. However, the auto‐regressive paths in the RI‐CLPM represent short‐term associations between one time point and the next, independent of trait‐like stability. Consequently, the RI‐CLPM does not allow for the effect of a stable predictor at baseline to cascade to later timepoints through the auto‐regressive paths, or from one trait to another via the cross‐lagged paths.

The main analysis was on parental warmth. Exploratory analyses were also conducted on home chaos and screen media use, the results of which are reported in the Supporting Information. Separate models were run on parental warmth data from adoptive mothers and adoptive fathers. Primary analyses were conducted using data from birth mothers because it is a larger sample than the sample of birth fathers. We conducted semi‐independent replications of these models using the smaller sample of birth fathers, providing an estimate of genetic effects that was not confounded by prenatal effects. Although the birth father sample is the largest ever recruited in a prospective parent–offspring adoption study, it has reduced statistical power compared to birth mother analyses. Although determining sample size requirements for complex structural equation models is not straightforward, generally a minimum sample size of 200 (Kline, 2016), or a sample size of five to 10 times the number of observed variables, is considered to be acceptable (Bentler & Chou, 1987; Nunnally, 1967). Our main analyses contained 29 observed variables, suggesting a sample size of >145–290. Thus, we anticipated that comparisons between results from birth mothers (*n* = 325) and birth fathers (*n* = 109) would be more focused on the magnitude of the path coefficients than on *p*‐values or confidence intervals. Based on recommendations by (Hu & Bentler, 1999), we used a combination rule according to which model fit was considered adequate if standardized root mean squared residual <.09 and root mean squared error of approximation <.06. The indirect effects were estimated using bootstrapping with 5000 repetitions (Bollen & Stine, 1990).

### Missing data

Sample sizes for the variables used in the main analyses are reported in Table 2. The primary source of missing data in models using birth mother data was children's phonological awareness subscale scores at age 6 years, measured using the TOPEL (available data for that subscale, *n* = 137). In birth father models, the primary source of missing data was missing information on birth father verbal IQ, measured using the WAIS information subscale (available data for that measure, *n* = 102). The data were not missing completely at random (MCAR [Little's MCAR *χ* ^2^(5635) = 6857, *p* < .01]). MCAR occurs when the probability of being missing is the same for all cases, and there is no systematic association between the missingness of the data and any other (observed or missing) values. As the data were not MCAR, we ran an attrition analysis using the Missing Value Analysis function in SPSS, which uses the *t*‐test procedure to compare group means and patterns of missingness in the data. This analysis revealed that the patterns of missingness for almost all (97%) of the variables used in our analyses were related to the observed values of other variables in the dataset. It was not possible to completely rule out the possibility that the data were missing not at random because that would require measuring the missing data (e.g., through following up non‐respondents). However, the observed patterns of missingness were consistent with the data being missing at random (MAR), which occurs when the missingness of a variable is systematically related to the observed but not unobserved data. Missing data were handled using full information maximum likelihood (FIML), which is suitable for data that are MAR and is of comparable performance to multiple imputation (Allison, 2003).

**TABLE 2:** Means, standard deviations, and sample sizes of study variables.

Variable	Birth parent general intellectual performance					
	Birth mother			Birth father		
	*n*	*M*	SD	*n*	*M*	SD
Wechsler Adult Intelligence Scale‐III, Information	323	9.56	2.59	102	10.65	2.88
Woodcock‐Johnson Tests of Achievement III, Letter‐Word Frequency	325	47.89	5.85	109	47.29	7.67
Woodcock‐Johnson Tests of Achievement III, Word Attack	325	49.80	7.53	109	46.60	7.80
Woodcock‐Johnson Tests of Achievement III, Reading Fluency	325	46.87	6.73	109	46.94	8.40
Woodcock‐Johnson Tests of Achievement III, Math Fluency	325	44.04	8.86	109	41.95	10.01

	Adoptive parent warmth					
	Adoptive mother			Adoptive father		
	*n*	*M*	SD	*n*	*M*	SD
4.5 years Let him/her know you really care about him/her	414	6.57	0.65	374	6.41	0.70
4.5 years Act loving and affectionate toward him/her	414	6.55	0.71	374	6.47	0.61
4.5 years Let your child know that you appreciate him/her, his/her ideas, or things he/she does	414	6.40	0.83	374	6.25	0.83
4.5 years Help him/her do something that was important to him/her	414	6.14	0.98	374	6.03	0.85
4.5 years Act supportive and understanding toward him/her	414	6.32	0.83	374	6.31	0.75
4.5 years Tell him/her you love him/her	399	6.79	0.56	361	6.71	0.61
6 years Let him/her know you really care about him/her	403	6.50	0.81	362	6.35	0.78
6 years Act loving and affectionate toward him/her	403	6.54	0.69	362	6.38	0.76
6 years Let your child know that you appreciate him/her, his/her ideas, or things he/she does	403	6.41	0.77	362	6.19	0.85
6 years Help him/her do something that was important to him/her	403	6.17	0.82	362	5.97	0.92
6 years Act supportive and understanding toward him/her	403	6.38	0.69	362	6.26	0.71
6 years Tell him/her you love him/her	403	6.78	0.57	362	6.63	0.68
7 years Let him/her know you really care about him/her	307	6.48	0.71	273	6.24	0.86
7 years Act loving and affectionate toward him/her	307	6.46	0.68	273	6.29	0.78
7 years Let your child know that you appreciate him/her, his/her ideas, or things he/she does	306	6.30	0.80	273	6.09	0.94
7 years Help him/her do something that was important to him/her	307	6.02	0.88	273	5.79	0.92
7 years Act supportive and understanding toward him/her	307	6.33	0.71	273	6.12	0.83
7 years Tell him/her you love him/her	307	6.75	0.54	273	6.56	0.76

	Child language and academic performance		
	*n*	*M*	SD
4.5 years Test of Preschool Early Literacy, Print Knowledge	291	106.80	13.39
4.5 years Test of Preschool Early Literacy, Definitional Vocabulary	288	104.30	9.94
4.5 years Test of Preschool Early Literacy, Phonological Awareness	137	97.76	15.66
6 years Wechsler Preschool and Primary Scale of Intelligence III	288	10.31	2.28
6 years Dynamic Indicators of Basic Early Literacy Skills, Initial Sound Fluency	141	58.48	30.22
6 years Dynamic Indicators of Basic Early Literacy Skills, Letter Naming Fluency	288	52.18	31.68
6 years Dynamic Indicators of Basic Early Literacy Skills, Phoneme Segmentation Fluency	293	33.84	27.87
6 years Dynamic Indicators of Basic Early Literacy Skills, Nonsense Word Fluency	293	46.19	32.58
7 years Woodcock‐Johnson Tests of Achieve Achievement III, Letter‐Word Frequency	336	56.29	9.23
7 years Woodcock‐Johnson Tests of Achieve Achievement III, Word Attack	334	53.91	10.80
7 years Woodcock‐Johnson Tests of Achieve Achievement III, Reading Fluency	334	55.22	7.51
7 years Woodcock‐Johnson Tests of Achieve Achievement III, Math Fluency	334	49.66	10.15

## RESULTS

Descriptive statistics (sample sizes, means, and standard deviations) for the variables used in the main analyses are presented in Table 2 and for the exploratory analyses are presented in Table S2. Birth parent scores on the Woodcock–Johnson test of achievement tended to be below the normative average of 50 (with the mean birth mother scores on the four subscales ranging from 46.87 to 49.80, and mean birth father scores ranging from 41.95 to 47.29), whereas adopted children had normed scores that tended to be higher than birth parent normed scores, and higher than the normative average of 50 (range of mean scores: 49.66–56.29). Mean scores of adopted child language at age 4.5 years, measured using the TOPEL, were close to the normative mean of 100 (ranging from 97.76 to 106.80), suggesting that their performance was close to the expected average for their age. Mean percentile scores at age 6 years on the DIBELS ranged from 33.84 to 58.25, which was within expected average range of 25–74. The adoptive parent self‐reported items on parental warmth were high: mean total scores (on a Likert scale from 1 to 7) ranged from 6.02 to 6.79 across all items and timepoints in the adoptive mother sample and ranged from 5.79 to 6.71 in the adoptive father sample.

### Direct and indirect effects of birth mother intellectual performance on adoptive parent warmth and children's language and academic performance

#### Adoptive mother

##### Evocative effects of birth mother intellectual performance on adoptive mother warmth

**FIGURE 2:**
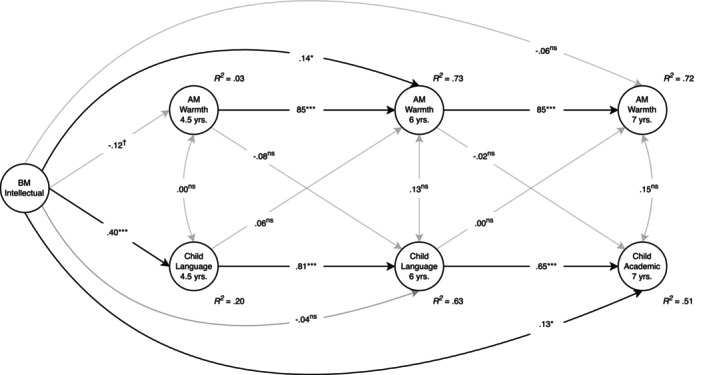
Longitudinal structural equation model examining the effects of birth mother intellectual performance on, and cross‐lagged associations between, adoptive mother warmth and Children's Language and Academic Performance. Model fit: *χ* ^2^(630) = 1346, *p* < .001, comparative fit index = .88, root mean squared error of approximation = .05, standardized root mean squared residual = .07. Standardized estimates reported. Adoption openness, child sex, and prenatal risk were included as covariates in the model. AM, adoptive mother; BM, birth mother. ***p < .001. *p < .05. ^†^ *p* < .1. ^ns^ *p* ≥ .1.

As displayed in Figure 2, there was a significant direct effect of birth mother intellectual performance (a proxy for genetic influences on children's intellectual and academic performance) on adoptive mother warmth when children were age 6 years (*β* = .14, 95% CI [.01, .27], *p* = .038) but not 4.5 years (*β* = −.12, 95% CI [−.26, .01], *p* = .063) or 7 years (*β* = −.06, 95% CI [−.17, .06], *p* = .361). The indirect effect of birth mother intellectual performance on adoptive mother warmth when children were age 7 years via warmth at 6 years was statistically significant (*β* = .12, 95% CI [.01, .23], *p* = .040). None of the other indirect or total effects of birth mother intellectual performance on adoptive mother warmth were statistically significant (see Table 3). The model accounted for 3% of the variance in adoptive mother warmth at 4.5 years, 73% of the variance at 6 years and 72% of the variance at 7 years. The large increase in the *R* ^2^ accounted for by the model at Ages 6 and 7 is primarily due to the high stability of adoptive mother warmth over time.

**TABLE 3:** Direct and indirect effects from the structural equation model displayed in Figure 2.

Test	Predictor	Outcome	Mediator/s	Paths	*β*	*p*
H1	BM intellectual	AM warmth 4.5 years	N/A	*a*	−.12 [−.26, .01]	.063
H1	BM intellectual	AM warmth 6 years	N/A	*b*	.14 [.01, .27]	.038
H1	BM intellectual	AM warmth 6 years	AM warmth 4.5 years	*ag*	−.11 [−.22, .01]	.069
H1	BM intellectual	AM warmth 6 years	AM warmth 4.5 years, child language 4.5 years	*b* + *ag* + *dm*	.06 [−.09, .21]	.445
H1	BM intellectual	AM warmth 7 years	N/A	*c*	−.06 [−.17, .06]	.361
H1	BM intellectual	AM warmth 7 years	AM warmth 4.5 years, AM warmth 6 years	*agh*	−.09 [−.18, .01]	.068
H1	BM intellectual	AM warmth 7 years	AM warmth 6 years	*bh*	.12 [.01, .23]	.040
H1	BM intellectual	AM warmth 7 years	AM warmth 4.5 years, AM warmth 6 years, child language 4.5 years, child language 6 years	*c* + *agh* + *akn* + *bh* + *dmh* + *din* + *en*	−.01 [−.15, .14]	.938
H2	BM intellectual	Child language 6 years	AM warmth 4.5 years	*ak*	.01 [−.01, .03]	.304
H2	BM intellectual	Child academic 7 years	AM warmth 4.5 years, child language 6 years	*akj*	.01 [−.01, .02]	.305
H2	BM intellectual	Child academic 7 years	AM warmth 6 years	*bl*	.00 [−.02, .01]	.784
H2	BM intellectual	Child academic 7 years	AM warmth 4.5 years, AM warmth 6 years	*agl*	.00 [−.01, .01]	.783
H2	BM intellectual	Child academic 7 years	AM warmth 4.5 years, AM warmth 6 years, child language 4.5 years	(*b* + *ag* + *dm*)*l*	.00 [−.01, .01]	.797
H3	BM intellectual	AM warmth 6 years	Child language 4.5 years	*dm*	.02 [−.03, .08]	.418
H3	BM intellectual	AM warmth 7 years	Child language 4.5 years, AM warmth 6 years	*dmh*	.02 [−.03, .07]	.418
H3	BM intellectual	AM warmth 7 years	Child language 6 years	*en*	.00 [.00, .00]	.979
H3	BM intellectual	AM warmth 7 years	Child language 4.5 years, child language 6 years	*din*	.00 [−.04, .04]	.979
H3	BM intellectual	AM warmth 7 years	Child language 4.5 years, child language 6 years, AM warmth 4.5 years	(*e* + *di* + *ak*)*n*	.00 [−.03, .03]	.979

*Note*: Values in square brackets indicate the 95% confidence intervals for the beta coefficients.

Abbreviations: AM, adoptive mother; BM, birth mother; H1, Hypothesis 1; H2, Hypothesis 2; H3, Hypothesis 3.

##### Mediation of birth mother effects on child language and academic performance via adoptive mother warmth

None of the cross‐lagged associations between adoptive mother warmth and child language or academic performance were statistically significant (see Figure 2). Nor were there any statistically significant indirect effects of birth mother intellectual performance on child language or academic performance, mediated via adoptive mother warmth (see Table 3).

##### Mediation of birth mother effects on adoptive mother warmth via child language

None of the indirect effects of birth mother intellectual performance on adoptive mother warmth, via child language, were statistically significant (see Table 3).

#### Adoptive father

##### Evocative effects of birth mother intellectual performance on adoptive father warmth

**FIGURE 3:**
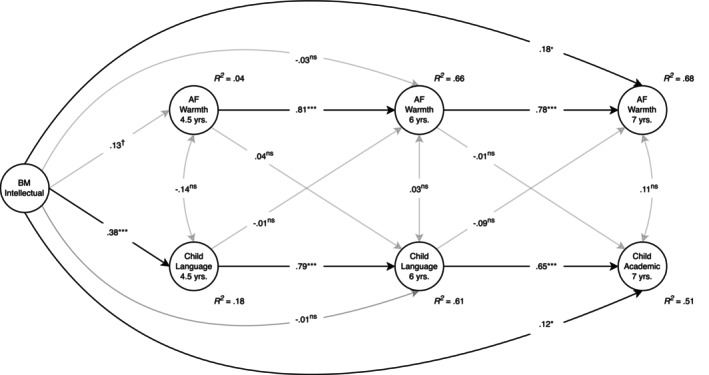
Longitudinal structural equation model examining the effects of birth mother intellectual performance on, and cross‐lagged associations between, adoptive father warmth and children's language and academic performance. Model fit: *χ* ^2^(630) = 1284, *p* < .001, comparative fit index = .88, root mean squared error of approximation = .05, standardized root mean squared residual = .07. Standardized estimates reported. Adoption openness, child sex, and prenatal risk were included as covariates in the model. AF, adoptive father; BM, birth mother. ***p < .001. *p < .05. ^†^ *p* < .1. ^ns^ *p* ≥ .1.

As displayed in Figure 3, there was a significant direct effect of birth mother intellectual performance on adoptive father warmth when children were 7 years of age (*β* = .18, 95% CI [.05, .31], *p* = .007). The direct effect of birth mother intellectual performance on adoptive father warmth was not statistically significant at age 4.5 years (*β* = .13, 95% CI [−.02, .28], *p* = .087) or 6 years (*β* = −.03, 95% CI [−.18, .11], *p* = .661). The total effect of birth mother intellectual performance on adoptive father warmth at age 7 years was statistically significant (*β* = .21, 95% CI [.06, .36], *p* = .006). None of the other indirect or total effects of birth mother intellectual performance on adoptive father warmth were statistically significant (see Table 4). The model accounted for 4% of the variance in adoptive father warmth at age 4.5 years, 66% of the variance in warmth at 6 years and 68% of the variance in warmth at 7 years. The large increase in the *R* ^2^ accounted for by the model at ages 6 and 7 is primarily due to the high stability of adoptive father warmth over time.

**TABLE 4:** Direct and indirect effects from the structural equation model displayed in Figure 3.

Test	Predictor	Outcome	Mediator/s	Paths	*β*	*p*
H1	BM intellectual	AF warmth 4.5 years	N/A	*a*	.13 [−.02, .28]	.087
H1	BM intellectual	AF warmth 6 years	N/A	*b*	−.03 [−.18, .11]	.661
H1	BM intellectual	AF warmth 6 years	AF warmth 4.5 years	*ag*	.11 [−.02, .23]	.092
H1	BM intellectual	AF warmth 6 years	AF warmth 4.5 years, child language 4.5 years	*b* + *ag* + *dm*	.07 [−.08, .22]	.340
H1	BM intellectual	AF warmth 7 years	N/A	*c*	.18 [.05, .31]	.007
H1	BM intellectual	AF warmth 7 years	AF warmth 4.5 years, AF warmth 6 years	*agh*	.08 [−.01, .18]	.088
H1	BM intellectual	AF warmth 7 years	AF warmth 6 years	*bh*	−.03 [−.14, .09]	.661
H1	BM intellectual	AF warmth 7 years	AF warmth 4.5 years, AF warmth 6 years, child language 4.5 years, child language 6 years	*c* + *agh* + *akn* + *bh* + *dmh* + *din* + *en*	.21 [.06, .36]	.006
H2	BM intellectual	Child language 6 years	AF warmth 4.5 years	*ak*	.01 [−.01, .03]	.560
H2	BM intellectual	Child academic 7 years	AF warmth 4.5 years, child language 6 years	*akj*	.00 [−.01, .02]	.560
H2	BM intellectual	Child academic 7 years	AF warmth 6 years	*bl*	.00 [.00, .00]	.887
H2	BM intellectual	Child academic 7 years	AF warmth 4.5 years, AF warmth 6 years	*agl*	.00 [−.01, .01]	.881
H2	BM intellectual	Child academic 7 years	AF warmth 4.5 years, AF warmth 6 years, child language 4.5 years	(*b* + *ag* + *dm*)*l*	.00 [−.01, .01]	.882
H3	BM intellectual	AF warmth 6 years	Child language 4.5 years	*dm*	.00 [−.06, .06]	.944
H3	BM intellectual	AF warmth 7 years	Child language 4.5 years, AF warmth 6 years	*dmh*	.00 [−.05, .05]	.944
H3	BM intellectual	AF warmth 7 years	Child language 6 years	*en*	.00 [−.01, .02]	.872
H3	BM intellectual	AF warmth 7 years	Child language 4.5 years, child language 6 years	*din*	−.03 [−.07, .01]	.178
H3	BM intellectual	AF warmth 7 years	Child language 4.5 years, child language 6 years, AF warmth 4.5 years	(*e* + *di* + *ak*)*n*	−.03 [−.07, .01]	.185

*Note*: Values in square brackets indicate the 95% confidence intervals for the beta coefficients.

Abbreviations: AF, adoptive father; BM, birth mother; H1, Hypothesis 1; H2, Hypothesis 2; H3, Hypothesis 3.

##### Mediation of birth mother effects on child language and academic performance via adoptive father warmth

None of the cross‐lagged associations between adoptive father warmth and child language or academic performance were statistically significant (see Figure 3). Nor were there any statistically significant indirect effects of birth mother intellectual performance on child language or academic performance, mediated via adoptive father warmth (see Table 4).

##### Mediation of birth mother effects on adoptive father warmth via child language

None of the indirect effects of birth mother intellectual performance on adoptive father warmth, via child language, were statistically significant (see Table 4).

### Direct and indirect effects of birth father intellectual performance on adoptive parent warmth and children's language and academic performance

#### Adoptive mother

##### Evocative effects of birth father intellectual performance on adoptive mother warmth

In the semi‐independent replication using birth father intellectual ability as a proxy for genetic influences, the positive effect on adoptive mother warmth when children were 6 years of age (which was significant in the birth mother model in Figure 2 was not significant, *β* = .07, 95% CI [−.15, .28], *p* = .530). As in the birth mother model, the direct effect of birth father intellectual performance on adoptive mother warmth was not significant at age 4.5 years (*β* = .10, 95% CI [−.18, .38], *p* = .473) or 7 years (*β* = .00, 95% CI [−.25, .25], *p* = .988). None of the other indirect or total effects of birth mother intellectual performance on adoptive father warmth were statistically significant. The model accounted for 3% of the variance in adoptive mother warmth at age 4.5 years, 72% of the variance in warmth at 6 years and 71% of the variance in warmth at 7 years. The large increase in the *R* ^2^ accounted for by the model at ages 6 and 7 is primarily due to the high stability of adoptive mother warmth over time.

##### Mediation of birth father effects on child language and academic performance via adoptive mother warmth

As in the birth mother model (displayed in Figure 2), in the birth father replication, none of the cross‐lagged associations between adoptive mother warmth and child language or academic performance were statistically significant. Nor were there any statistically significant indirect effects of birth father intellectual performance on child language or academic performance, mediated via adoptive mother warmth.

##### Mediation of birth father effects on adoptive mother warmth via child language

None of the indirect effects of birth father intellectual performance on adoptive mother warmth, via child language, were statistically significant.

#### Adoptive father

##### Evocative effects of birth father intellectual performance on adoptive father warmth

In the birth father replication, the effect on adoptive mother warmth when children were age 7 years, which was significant in the birth mother model (Figure 3), was not statistically significant (*β* = .14, 95% CI [−.08, .36], *p* = .224). As in the birth mother model (Figure 3), in the birth father model the effects on warmth at ages 4.5 (*β* = −.01, 95% CI [−.30, .28], *p* = .949) and 6 years (*β* = −.22, 95% CI [−.53, .08], *p* = .151) were not significant. None of the other indirect or total effects of birth father intellectual performance on adoptive father warmth were statistically significant. The model accounted for 2% of the variance in adoptive father warmth at age 4.5 years, 69% of the variance in warmth at 6 years and 66% of the variance in warmth at 7 years. The large increase in the *R* ^2^ accounted for by the model at ages 6 and 7 is primarily due to the high stability of adoptive father warmth over time.

##### Mediation of birth father effects on child language and academic performance via adoptive father warmth

As in the birth mother model (displayed in Figure 3), in the birth father replication, none of the cross‐lagged associations between adoptive father warmth and child language or academic performance were statistically significant. Nor were there any statistically significant indirect effects of birth father intellectual performance on child language or academic performance, mediated via adoptive father warmth.

##### Mediation of birth father effects on adoptive father warmth via child language

None of the indirect effects of birth father intellectual performance on adoptive father warmth, via child language, were statistically significant.

### Exploratory analyses on household chaos and screen media use

Results from exploratory analyses examining household chaos and screen media use are reported in the Supporting Information. There was no evidence of evocative effects of genetic influences underlying children's intellectual performance on household chaos or screen media use. There were no significant indirect effects of birth parent intellectual ability on child language or academic performance via adoptive parent warmth or of birth parent intellectual ability on adoptive parent warmth via child language. However, there was an expected statistically significant negative association between children's verbal performance at age 4.5 years and screen media use at 6 years, as well as an unexpected statistically significant positive association between screen media use at age 4.5 years and children's language at age 6 years.

### Adoption openness sensitivity analysis

We conducted a sensitivity analysis, including the interaction term (birth parent intellectual ability × adoption openness) in the SEMs. As reported in detail in the Supporting Information, the results remained largely unchanged.

## DISCUSSION

This study leveraged the parent‐offspring adoption design to examine the potential role of *r*GE in the development of children's intellectual and academic abilities. The results demonstrated that birth mother intellectual performance (used as a proxy for genetic influences on children's intellectual and academic outcomes) predicted adoptive mother warmth when children were 6 and 7 years of age, but not 4.5 years, and adoptive father warmth at age 7 years, but not 4.5 or 6 years. These findings are partially consistent with the first hypothesis that there would be evocative or active effects on the caregiving environment of genetic influences underlying children's academic ability, and evidence from twin and polygenic score research of evocative *r*GE in early cognitive development (Tucker‐Drob & Harden, 2012; Wertz et al., 2020). However, results from the birth mother models were not fully replicated in the analyses using data from birth fathers as the proxy for genetic influences.

Contrary to the second and third hypotheses that evocative or active effects on the caregiving environment would mediate genetic effects on academic outcomes and that language would be a mechanism through which genetic influences would evoke parenting differences, there was no evidence of bidirectional associations between adoptive parent warmth and children's language and academic outcomes or indirect effects via parenting or child language, respectively. Although these findings indicate that adoptive parents might parent their children differently depending on their children's intellectual and academic performance‐associated genetics, they do not demonstrate a mediating influence of these parenting differences on children's academic test performance in middle childhood. The only statistically significant effects on children's language and academic test outcomes were the effects of birth parent intellectual performance reported in earlier work (Austerberry, Fearon, et al., 2022). Furthermore, despite this earlier work indicating that language appears to be an early manifestation of genetic influences on later academic outcomes, our findings do not indicate that early language evokes differences in parental warmth. Consequently, the present findings failed to uncover evidence to support the Dickens and Flynn (2001) hypothesis that increasing heritability of intellectual performance across the lifespan is produced through a process of amplification via mechanisms of reciprocal causation between an individual's genetics and the environmental influences they evoke or actively select over time, or the hypothesis that early language is a mechanism that evokes differences in parenting.

Based on the well‐established associations between positive parenting and children's intellectual and academic outcomes (Fan & Chen, 2001; Lugo‐Gil & Tamis‐LeMonda, 2008; Madigan et al., 2019; Wertz et al., 2020), it is surprising that parental warmth did not predict children's language or academic performance. This lack of association suggests that previous findings may be attributable to passive *r*GE. Indeed, Wertz et al. (2020) demonstrated in the E‐Risk Study, first, that parent and child education polygenic scores were each pleiotropic: associated with positive parenting *and* children's academic performance. Second, when they partialled out the effects of children's polygenic scores, the association between parenting and children's educational attainment became attenuated, indicating some genetic confounding. In contrast to our findings, although the association reduced in size, it remained statistically significant after the polygenic score was partialled out. However, as Wertz et al. (2020) discuss, based on the limitations of polygenic scores discussed in greater detail below, unlike the adoption design, their methods do not rule out the possibility of passive *r*GE and likely only control for a small proportion of the genetic confounding. Another possible explanation for the lack of association between parental warmth and academic test performance is that warmth may be less important for academic performance than other dimensions of positive parenting, particularly as children begin formal schooling. Indeed, Lugo‐Gil and Tamis‐LeMonda (2008) and Wertz et al. (2020) both used more global measures of positive parenting, the former combining measures of maternal supportiveness, sensitivity, positive regard, and cognitive stimulation and the latter combining measures of warmth, sensitivity and reverse‐coded negative parenting. Furthermore, the meta‐analysis of parenting and academic achievement in childhood and adolescence by Fan and Chen (2001) found that parental involvement was associated with children's academic achievement (*r* = .30) and that within the domain of parental involvement the subdomain with the largest effect size was parents' aspiration and expectation for children's educational achievement (*r* = .40). In the series of meta‐analyses by Madigan et al. (2019), the pooled effect was larger for the association between sensitive‐responsive parenting and child language (*r* = .27) than for the association between parental warmth and child language (*r* = .16). Consequently, the lack of association between parenting and language or academic performance may represent an inappropriate choice of parenting measure. It may also reflect limited generalizability of the present findings due to use of an unrepresentative sample, which is discussed in the limitations section below.

In exploratory analyses of home chaos and screen media use, there were no significant effects of birth parent intellectual performance on household chaos or children's screen media use, the latter based on a composite of items on television watching and video gaming. In other words, there was no evidence of evocative or active *r*GE in these analyses. Nor were there any significant cross‐lagged associations between household chaos and children's language or academic test performance. However, there were two (out of four) significant cross‐lagged associations in the model examining screen media use: as hypothesized, children's language at age 4.5 years was negatively associated with screen media use at age 6 years; unexpectedly, screen media use at age 4.5 years was positively associated with language performance at age 6 years. Although this latter finding runs counter to the general trend in the literature that screen time is negatively associated with intellectual and academic performance throughout childhood and adolescence (Adelantado‐Renau et al., 2019), evidence also suggests that high‐quality and parent‐monitored screen time is positively associated with language and academic outcomes (Adelantado‐Renau et al., 2019; Madigan et al., 2020). Consequently, this negative association in our sample may reflect the possibility that a high socioeconomic status (SES) sample of parents who have had to pass stringent tests determining their suitability to adopt are more likely to monitor the types of screen time their children are exposed to than a representative sample would. Screen use was also relatively low in the EGDS sample (*M* range: 1.4 and 2.4 h per day of television watching or computer games), potentially limiting generalizability of our findings. Finally, although our findings indicate that screen media use at age 4.5 years may positively influence language performance, they do not suggest that screen media use in the home mediates genetic influences on academic test performance. As in the analyses of parental warmth and household chaos, analyses of screen media use yielded no evidence to support the Dickens and Flynn (2001) hypothesis that evocative or active *r*GE influences the development of intellectual and academic skills.

### Limitations and future directions

Our research is the first to examine evocative effects of genetic factors associated with intellectual or academic development using data from an adoption study, providing a powerful control against passive *r*GE, which is the phenomenon that occurs when the genes children inherit from their biological parents are correlated with the environment they are raised in, making it challenging to disentangle genetic from environmental effects when studying biological families (Plomin et al., 1977; Scarr & McCartney, 1983). The assumption that passive *r*GE is ruled out by the adoption design depends on the environments of adoptees not being influenced by their birth parents. Early placement of EGDS adoptees (on average 6 days postpartum) reduced the likelihood of this assumption being violated. However, there are two potential threats to this presumption that are important to consider. First, any ongoing contact between adoptees and their birth parents introduces the possibility of birth parents influencing adoptees' environments. We attempted to control for this potential confound by including a composite of birth and adoptive parent ratings of adoption openness as a covariate in our analyses, as well as the interaction term (birth parent intellectual ability × adoption openness) in additional sensitivity analyses. The second potential threat to the assumption that the adoption design rules out passive *r*GE is that passive *r*GE could occur if any aspects of the prenatal environment are correlated with the genes that birth parents pass on to their children. We controlled for this potential confound by including a measure of prenatal risk as a covariate in our models and replicating our analyses in the birth father sample. Birth father analyses are a robust control for prenatal effects only if birth fathers do not indirectly affect the prenatal environment by, for example, contributing to home dynamics or stress levels of the mother. The low rates of birth mother cohabitation (6.1%) in our sample plausibly reduce the chances of indirect effects of birth fathers on the fetal environment (Leve et al., 2019).

Overall, it is a strength of the present study that we were able to run two sets of analyses: the main analysis in a sample of birth mothers and a quasi‐independent replication in a sample of birth fathers. This feature is a particular strength as fathers are so under‐researched relative to mothers in developmental research. However, the birth father analyses were not fully independent because most measures of birth mother and birth father intellectual performance were associated, suggesting the possibility of assortative mating, confounding, or partner interaction effects (Austerberry, Fearon, et al., 2022). Despite being the largest sample of birth fathers ever recruited in a prospective parent–offspring adoption study, the birth father analyses were limited by their small sample size, resulting in a lack of statistical power to accurately estimate the influence of birth father intellectual performance. This lack of power may explain why the findings from the birth mother models were not fully replicated in the birth father analyses. However, we were not able to rule out the possibility that the lack of robust replication signaled either spurious results in the birth mother models, or prenatal (rather than genetic) effects being detected in associations between birth mother intellectual performance and adoptive parent parenting. Although the likelihood of the latter is probably reduced by the inclusion of a prenatal risk covariate, replication of our methods using larger samples of birth fathers is needed before decisive conclusions can be drawn.

Although the adoption design has notable strengths, an inherent limitation is that there is relatively little variation in family income and parental education in adoptive families compared to the general population. The adoptive families in our sample had a higher SES than the birth parents in the study and the general population of the United States, potentially biasing the results (Leve et al., 2013). Thus, it remains to be seen whether our findings would replicate in lower SES families. SES may causally influence parenting (Akee et al., 2010) and appears to moderate genetic effects on academic outcomes, enhancing the expression of genetic contributions in higher SES samples at the expense of environmental effects and enhancing the expression of environmental contributions in lower SES samples at the expense of genetic effects (Scarr‐Salapatek, 1971; Tucker‐Drob & Bates, 2015; Turkheimer et al., 2003). Research suggests that adoptees in the United States and United Kingdom perform better than expected academically based on their preadoption intelligence scores, education polygenic scores or comparisons with their biological relatives after being adopted (Cheesman et al., 2020; Duyme et al., 1999; Kendler et al., 2015), indicating mediation or moderation of genetic effects on academic performance by differences in caregiving environments (or the wider social conditions that are associated with them).

There is also evidence from the United States and United Kingdom that trajectories of language and academic development are not the same for different ethnic groups, and this effect may be explained by differences in psychosocial, family, and home environments (Saccuzzo et al., 1992; Zilanawala et al., 2016). Although almost half of the adoptees in the EGDS sample were multiracial, Black or African American, or Latinx, over 90% of the adoptive parents in the study were non‐Latinx White and the study was U.S.‐based, adding to the literature on samples from Western, educated, industrialized, rich, and democratic populations, who, despite making up approximately 12% of the world's population, are the subject of the vast majority of findings published in top psychology research journals (Arnett, 2008). Replication of our methods in different populations is needed to address this stark inequity and before we can assume that our results generalize.

Finally, there are three potential limitations concerning measurement and the operationalization of the analyzed constructs. First, there may be limits to the extent to which the measures analyzed were suitable for testing the Dickens and Flynn (2001) hypothesis. We focused the analyses primarily on parental warmth on the basis that prior research had demonstrated not only that parental positivity and warmth appear to predict better intellectual and academic outcomes in children but also that children's intellectual abilities, or genetic propensities linked to intellectual and academic abilities, appear to positively predict the warmth and positivity of the parenting they receive (Lugo‐Gil & Tamis‐LeMonda, 2008; Madigan et al., 2019; Wertz et al., 2020). However, another dimension of parenting that prior evidence suggests may be evoked by children's cognition and education linked to genetic propensities is cognitively stimulating parenting (Tucker‐Drob & Harden, 2012; Wertz et al., 2020), which (as discussed in detail above) may also be important for children's educational development. There were no suitable data available in the pre‐existing dataset used for the present analyses to test the Dickens and Flynn (2001) hypothesis using measures of parental cognitive stimulation at 4.5–7 years. However, it would be of interest for future work to incorporate such measures. Furthermore, Dickens and Flynn (2001) describe a multiplicity of many individual and social factors evoked or selected over time, including activities undertaken during leisure time, intellectual quality of social interactions, intellectual demands at school, and intellectual complexity of work. Individually, these factors do not necessarily exert a large effect but cumulatively may result in substantial change in intellectual ability across development. Consequently, a fairer test of the Dickens and Flynn (2001) hypothesis would incorporate many varied measures over time, rather than focus specifically on parenting during a narrow age range. Second, as our results rely on self‐reports by parents of their parenting and the caregiving environment, they are vulnerable to reporter bias and ceiling effects, which occur when a large proportion of respondents score near the upper limit of a scale so that variance is not measured above a certain level. In the present sample, most adoptive parents rated their parenting as “almost always” or “always” warm, resulting in low variability in the responses. Consequently, future research should incorporate observational measurement to potentially increase variability and reduce both informant and method bias. Third, we did not use direct genetic measures and instead relied on measures of birth parent traits as indirect proxies for the genetic influences. However, it remains open for debate which behavior genetic methods best capture the full contribution of genetic influences, as there is a discrepancy (known as “missing” heritability) between estimates from genome‐wide analyses and those relying on family data such as adoption or, more commonly, twin studies. For example, in the most recently published genome‐wide association study of educational attainment, a polygenic index explained 12%–16% of the variance in educational attainment (Okbay et al., 2022)—around one‐third of the size of the 43% heritability estimate reported in a recent analysis of a pooled sample of 28 twin cohorts (Silventoinen et al., 2020). Consequently, it remains helpful to continue triangulating findings from studies using different research designs and methods. Our work contributes to this effort as evocative or active *r*GE in intellectual development had only ever been examined using the twin design and genome‐wide polygenic scores, and our research is the first to examine these mechanisms in intellectual and academic development using an adoption study and birth parent trait status as a proxy for genetic influences.

## CONCLUSION

In this first empirical test of the Dickens and Flynn (2001) hypothesis and examination of evocative or active *r*GE in academic development in an adoption sample, we found no evidence to support the Dickens and Flynn (2001) hypothesis but some evidence of evocative effects of genetic influences underlying children's intellectual and academic development on parental warmth. While these effects did not seem to be evoked by differences in early language performance and did not mediate associations between genetic influences and middle‐childhood academic outcomes, they nonetheless converge with findings from twin and polygenic score research in suggesting that parents may parent their children differently depending on their children's genetic predispositions for academic attainment.

## FUNDING INFORMATION

Data collection and a portion of David Reiss's, Leslie D. Leve's, Jenae M. Neiderhiser's, and Jody M. Ganiban's effort were supported in part by R01 HD042608 from the Eunice Kennedy Shriver National Institute of Child Health and Human Development and the National Institute on Drug Abuse (NIDA), NIH, U.S. PHS (PI Years 1–5: David Reiss, MD; PI Years 6–10: Leslie Leve, PhD); R01 DA020585 and from the NIDA, the National Institute of Mental Health (NIMH) and Office of Behavioral and Social Sciences Research (OBSSR), NIH, U.S. PHS (PI: Jenae Neiderhiser, PhD); R01 MH092118 from the NIMH, NIH, U.S. PHS (PIs Jenae Neiderhiser, PhD and Leslie Leve, PhD); R01 DA035062 from the NIDA, NIH, U.S. PHS (PI Leslie Leve, PhD); and UH3OD023389 from the Office of the Director, NIH. Chloe Austerberry's time on this work was supported by a UK Research and Innovation (UKRI) Economic and Social Research Council (ESRC) Studentship awarded by the UCL, Bloomsbury and East London Doctoral Training Partnership (ES/P000592/1). The content is solely the responsibility of the authors and does not necessarily represent the official views of the NIH or UKRI.

## CONFLICT OF INTEREST STATEMENT

No other conflicts of interest are declared by the authors.

## ETHICS STATEMENT

Ethical approval was obtained from institutional review boards at the University of Oregon (Protocol number: 0304201400) and The Pennsylvania State University (Submission ID: CR00007591).

## Supporting information


Appendix S1.

## Data Availability

The data, analytic code, and materials necessary to reproduce the analyses presented here are not publicly accessible, however, the analytic code is available from the first author on request. The analyses presented here were preregistered. The preregistration is available at the following URL: https://osf.io/pz67m.
