# Supplementary material for: Evocative effects on the early caregiving environment of genetic factors underlying the development of intellectual and academic ability
Source: Child Dev. 2024 Jul 30;95(6):2082–101. doi: 10.1111/cdev.14142 (PMC11579646; doi:10.1111/cdev.14142)
Supplement: Supplementary file 1 — Appendix S1. [file CDEV-95-2082-s001.docx]

**Supplement**

Exploratory Factor Analysis

Prior to hypothesis testing, we examined the factor structure of several parenting measures administered to adoptive parents, to determine which items to include in the latent variables constructed for the main analysis. We conducted the exploratory analyses using data from a random split-half subsample (*n* = 264) at 4.5, 6 and 7 years. The factorability of 39 parenting items was examined. Sixteen of the 39 items were from the Alabama Parenting Questionnaire (APQ) (Frick, 1991), 10 of which were from the ‘Involvement’ subscale of the APQ (measuring parental involvement in the child’s daily routine and activities) and six of which were from the ‘Positive Parenting’ subscale of the APQ (measuring the extent to which the parent provides positive feedback or rewards for their child). Six of the 39 items were from the ‘Warmth’ subscale of the Iowa Family Interaction Rating Scales (IOWA) (Melby & Conger, 2001), measuring a parent’s warmth towards their child. Eleven of the 39 items were questions on the Home Literacy Environment (HLE) (Johnson, Martin, Brooks-Gunn, & Petrill, 2008; Niklas & Schneider, 2013), measuring family-level HLE characteristics (such as whether the family uses a library card and number of magazines in the home) as well as child-level HLE characteristics (such as how many books the child owns and how much television they watch). Six items were from the Chaos, Hubbub, and Order Scale (CHAOS) (Matheny, Wachs, Ludwig, & Phillips, 1995), measuring quietness and order of the home.

A correlation matrix of the 39 parenting items revealed that all but 10 items were sufficiently positively or negatively correlated (≥ .3 or ≤ −.3) with at least one other item at 4.5 and 6 years, and all but 11 items were correlated at 7 years, suggesting reasonable factorability. The items with poor factorability (correlated < .3 or > −.3) were removed from each wave of data. We then used version 2.2.9 of the psych package in R (Revelle, 2022) to conduct Bartlett’s Test of Sphericity, which tests whether variables are suitable for data reduction techniques (such as exploratory factor analysis [EFA]) by comparing the observed correlation matrix to the identity matrix. The result was significant at each timepoint, suggesting the remaining parenting variables were suitable for EFA: 4.5 years, χ^2^(406) = 2254, *p* < .001; 6 years, χ^2^(406) = 2164, *p* < .001; 7 years, χ^2^(378) = 1972, *p* < .001. To determine how many factors to extract, at each timepoint we created a scree plot of successive eigenvalues from a principal components analysis of the parenting items, using version 2.2.9 of the psych package in R (Revelle, 2022). A widely used decision rule is to retain the factors to the left of the point of inflection in a scree plot of eigenvalues (Cattell, 1966). In the scree plots, the points of inflection were between components 2 and 4, indicating between a one-factor and three-factor solution.

Using the R packages psych (version 2.2.9) (Revelle, 2022) and GPArotation (Bernaards & Jennrich, 2005), we ran a three-factor maximum likelihood factor analyses with oblique (oblimin) rotation (as we were expecting the factors to correlate). The results from this analysis are displayed in Table S1. The items that cluster on the same factor suggest that factor 1 represents positive/warm parenting, factor 2 represents household and child screen media use, and factor 3 represents household chaos. Between them, the 3 factors explained 39% of the total variance at 4.5 years, 37% at 6 years, and 38% at 7 years. Factor 1 explained 22% of the variance at each timepoint. Factor 2 explained 9% of the variance at 4.5 and 7 years, and 7% at 6 years. Factor 3 explained 8% of the variance at 4.5 years and 7% at 6 and 7 years. At all timepoints factor 1 was very weakly negatively correlated with factor 2 (4.5 years, *r* = −.02; 6 years, *r* = −.01; 7 years, *r* = −.06) and weakly negatively correlated with factor 3 (4.5 years, *r* = −.20; 6 years, *r* = −.23; 7 years, *r* = −.19), and Factors 2 and 3 were very weakly positively correlated (4.5 years, *r* = .04; 6 years, *r* = .07; 7 years, *r* = .13). All items from the IOWA, and most (but not all) items from the Alabama had high factor loadings on factor 1. Evidence suggests that more indicators per factor is not necessarily better and using too many indicators per factor can create bias in the model chi-square statistic, requiring an increase in sample size to compensate for the increasing model size (Koran, 2020). Consequently, for the main analyses, we decided to drop the items from the APQ and retain the 6 items from the IOWA as indicators in a latent variable measuring positive parenting. The 3 HLE items and single item from the CHAOS that had the highest factor loadings on factor 2 were retained for the latent variable measuring screen media use. The 3 items from the CHAOS that had the highest factor loadings on factor 3 were used as indicators in the latent variable measuring household chaos. Further information on the parenting warmth measures used in the main analysis is reported in the measures section of the main manuscript. Additional information on the measures of household chaos and screen media use constructed for the exploratory analyses is reported below.

**Construction of Cross-Lagged Panel Models**

The full cross-lagged panel model (CLPM) was built in several steps: First, we constructed an autoregressive model examining parental warmth (and in the exploratory analyses, household chaos and screen media use) when children were 4.5, 6, and 7 years old. In the autoregressive model warmth (or chaos/screen use) at 7 years was regressed on warmth (or chaos/screen use) at 6 years, which was regressed on warmth (or chaos/screen use) at 4.5 years. Second, we introduced birth parent general intellectual and academic performance (a proxy for genetic influences on children’s intellectual and academic abilities) as a predictor in the autoregressive models, examining whether the birth parent general intellectual and academic performance latent variable predicted adoptive parent warmth (or chaos/screen use) at 4.5, 6, and 7 years. Third, we constructed a CLPM, combining the parenting autoregressive models and genetic predictor with an autoregressive model examining children’s language and academic test performance reported in previous work (Austerberry et al., 2022). The following cross-lagged associations between adoptive parental warmth (or chaos/screen use) and children’s language and academic test performance were included in the model: child academic test performance at 7 years old was regressed on parental warmth (or chaos/screen use) at 6 years old; child language ability at 6 years old was regressed on warmth (or chaos/screen use) at 4.5 years old; parental warmth (or chaos/screen use) at 7 years old was regressed on child language at 6 years old; and warmth (or chaos/screen use) at 6 years old was regressed on language at 4.5 years old. Additionally, language and warmth (or chaos/screen use) were allowed to covary at each timepoint.

Table S1

*Results from Exploratory Factor Analysis of Parenting Items*

|  | Oblimin Rotated Factor Loadings | | | | | | | | |
| --- | --- | --- | --- | --- | --- | --- | --- | --- | --- |
|  | Positive/Warm Parenting | | | Household and Child Screen Media Use | | | Household Chaos | | |
| Item | 4.5 yrs. | 6 yrs. | 7 yrs. | 4.5 yrs. | 6 yrs. | 7 yrs. | 4.5 yrs. | 6 yrs. | 7 yrs. |
| You can’t hear yourself think in our home. | .01 | .02 | -.02 | -.03 | .00 | -.06 | **.83** | **.84** | **.83** |
| It’s a real zoo in our home. | .02 | .04 | -.01 | .01 | -.01 | .10 | **.90** | **.85** | **.81** |
| We are usually able to stay on top of things. | .11 | .08 | .09 | -.04 | -.17 | .02 | -.36 | -.17 | -.28 |
| There is usually a television turned on somewhere in our home. | .00 | .03 | .00 | **.58** | **.46** | **.52** | .19 | .02 | .14 |
| The atmosphere in our house is calm. | .06 | .21 | .01 | -.03 | .00 | .00 | **-.75** | **-.59** | **-.67** |
| Weekdays (Monday-Friday): On average, how many hours per day does your child watch TV or play video games? | .04 | .08 | .04 | **.69** | .36 | **.57** | .02 | .11 | .06 |
| Saturday: On average, how many hours per day does your child watch TV or play video games? | -.03 | .01 | .01 | **.94** | **.88** | **.98** | -.03 | .01 | .01 |
| Saturday: On average, how many hours per day does your child watch TV or play video games? | .02 | -.01 | -.03 | **.84** | **.92** | **.85** | -.02 | -.03 | -.04 |
| Let him/her know you really care about him/her | **.57** | **.71** | **.76** | -.03 | .01 | -.05 | -.10 | -.05 | .14 |
| Act loving and affectionate toward him/her | **.55** | **.70** | **.78** | -.08 | .05 | -.02 | -.06 | .04 | -.06 |
| Let your child know that you appreciate him/her, his/her ideas, or things he/she does | **.57** | **.82** | **.85** | -.06 | -.01 | .06 | -.05 | .02 | -.02 |
| Help him/her do something that was important to him/her | **.62** | **.75** | **.75** | -.10 | .01 | .01 | -.02 | .04 | -.08 |
| Act supportive and understanding toward him/her | **.46** | **.69** | **.63** | .02 | .06 | .03 | -.15 | .02 | -.08 |
| Tell him/her you love him/her | **.56** | **.65** | **.65** | .02 | -.01 | .02 | .01 | .02 | .07 |
| You have a friendly talk with your child. | .27 | **.47** | **.50** | -.05 | .00 | .03 | -.14 | -.07 | -.06 |
| You let your child know when he/she is doing a good job with something. | **.65** | **.55** | **.56** | .06 | -.09 | .07 | -.15 | .02 | -.04 |
| You volunteer to help with special activities that your child is involved in. | **.48** | .39 | .35 | -.03 | -.10 | -.15 | .10 | -.03 | .02 |
| You reward or give something extra to your child for obeying you or behaving well. | **.52** | **.46** | **.41** | -.05 | -.12 | -.02 | -.12 | -.09 | -.07 |
| You ask your child about his/her day in school. | **.52** | **.49** | **.40** | -.06 | .02 | -.09 | .03 | .08 | .05 |
| You help your child with his/her homework. | **.42** | .35 | .20 | .10 | -.08 | .03 | .01 | -.04 | -.01 |
| You compliment your child when he/she does something well. | **.65** | **.49** | **.43** | .10 | .09 | -.04 | -.06 | -.20 | -.02 |
| You ask your child what his/her plans are for the coming day. | **.48** | **.52** | **.41** | -.07 | -.04 | -.04 | .08 | -.07 | .18 |
| You drive your child to a special activity. | **.59** | .39 | **.43** | .01 | -.21 | -.05 | .09 | -.03 | -.05 |
| You praise your child if he/she behaves well. | **.65** | **.55** | **.48** | .11 | .09 | .08 | .00 | -.05 | -.06 |
| You hug or kiss your child when he/she has done something well. | **.61** | **.60** | **.63** | .14 | .08 | -.04 | .11 | .09 | .07 |
| You talk to your child about his/her friends. | **.60** | **.47** | **.54** | -.07 | -.05 | -.08 | .03 | -.12 | .03 |
| Your child helps plan family activities. | **.41** | **.45** | **.42** | -.07 | -.07 | -.07 | .08 | .03 | .03 |
| You attend PTA meetings, parent/teacher conferences, or other meetings at your child’s school. | **.50** | .21 | .11 | -.15 | -.12 | -.11 | .09 | -.13 | -.10 |
| You tell your child that you like it when he/she helps out around the house. | **.59** | **.47** | NA | .01 | -.15 | NA | .09 | -.04 | NA |
| Eigenvalues | 6.25 | 6.52 | 6.15 | 2.53 | 2.15 | 4.40 | 2.41 | 2.00 | 2.06 |
| Proportion of variance | .22 | .22 | .22 | .09 | .07 | .09 | .08 | .07 | .07 |
| *Note.* Factor loadings over .40 appear in bold | | | | | | | | | |

Descriptive Statistics in Exploratory Analyses

The descriptive statistics (sample sizes, means and standard deviations) for the measures of the caregiving environment included in the exploratory analyses on household chaos and household and child screen media use are reported in Table S2. Mean scores of household chaos ranged from 2.07 to 2.47, which was between 2, “somewhat untrue”, and 3, “not really true or untrue”, when adoptive parents rated statements describing a chaotic home environment. Mean adoptive parent-rated estimates of the amount of time their children spend watching TV or playing computer games ranged from 1.42 to 2.43 hours per day. Mean adoptive parent ratings of whether “there is usually a TV on in our home” ranged from 2.55 to 2.62, which was between 2, “somewhat untrue”, and 3, “not really true or untrue”.

Table S2

*Means, Standard Deviations and Sample Sizes of Variables in Exploratory Analyses*

|  | Household Chaos | | |
| --- | --- | --- | --- |
| Variable | *n* | *M* | *SD* |
| 4.5 yrs. You can’t hear yourself think in our home | 426 | 2.24 | 0.98 |
| 4.5 yrs. It’s a real zoo in our home | 426 | 2.13 | 0.97 |
| 4.5 yrs. The atmosphere in our house is calm (reverse scored) | 426 | 2.43 | 0.90 |
| 6 yrs. You can’t hear yourself think in our home | 409 | 2.20 | 0.94 |
| 6 yrs. It’s a real zoo in our home | 409 | 2.07 | 0.93 |
| 6 yrs. The atmosphere in our house is calm (reverse scored) | 409 | 2.40 | 0.86 |
| 6 yrs. You can’t hear yourself think in our home | 412 | 2.27 | 0.98 |
| 6 yrs. It’s a real zoo in our home | 412 | 2.17 | 0.97 |
| 6 yrs. The atmosphere in our house is calm (reverse scored) | 412 | 2.47 | 0.86 |
|  | Household and Child Screen Media Use | | |
| Variable | *n* | *M* | *SD* |
| 4.5 yrs. On average, how many hours per day does your child watch television or play video games on weekdays? | 425 | 1.67 | 1.16 |
| 4.5 yrs. On average, how many hours per day does your child watch television or play video games on Saturday? | 425 | 2.12 | 1.16 |
| 4.5 yrs. On average, how many hours per day does your child watch television or play video games on Sunday? | 425 | 1.94 | 1.16 |
| 4.5 yrs. There is usually a television turned on somewhere in our home | 426 | 2.62 | 1.30 |
| 6 yrs. On average, how many hours per day does your child watch television or play video games on weekdays? | 263 | 1.42 | 1.89 |
| 6 yrs. On average, how many hours per day does your child watch television or play video games on Saturday? | 263 | 2.19 | 1.07 |
| 6 yrs. On average, how many hours per day does your child watch television or play video games on Sunday? | 263 | 1.92 | 1.13 |
| 6 yrs. There is usually a television turned on somewhere in our home | 409 | 2.58 | 1.25 |
| 7 yrs. On average, how many hours per day does your child watch television or play video games on weekdays? | 311 | 1.42 | 0.99 |
| 7 yrs. On average, how many hours per day does your child watch television or play video games on Saturday? | 311 | 2.43 | 1.20 |
| 7 yrs. On average, how many hours per day does your child watch television or play video games on Sunday? | 311 | 2.30 | 1.22 |
| 7 yrs. There is usually a television turned on somewhere in our home | 412 | 2.55 | 1.27 |

**Direct and Indirect Effects of Birth Mother Intellectual Performance on Household Chaos and Children’s Language and Academic Performance**

**Evocative Effects of Birth Mother Intellectual Performance on Household Chaos.** As displayed in Figure S1, there were no significant direct effects of birth mother intellectual performance (a proxy for genetic influences) on household chaos (composite of adoptive mother and adoptive father ratings) at 4.5 years old (β = −0.08, 95% CI [−0.25, 0.10], *p* = .398), 6 years old (β = 0.06, 95% CI [−0.09, 0.21], *p* = .425), or 7 years old (β = −0.04, 95% CI [−0.18, 0.10], *p* = .560). Nor were there any statistically significant indirect or total effects of birth mother intellectual performance on household chaos (see Table S3). The model accounted for 3% of the variance in household chaos at 4.5 years, 87% of the variance in chaos at 6 years and 87% of the variance in chaos at 7 years. The large increase in the R^2^ accounted for by the model at ages 6 and 7 is primarily due to the high stability of household chaos over time.

**Mediation of Birth Mother Effects on Child Language and Academic Performance via Household Chaos.** None of the cross-lagged associations between household chaos and child language or academic performance were statistically significant (see Figure S1). Nor were there any statistically significant indirect effects of birth mother intellectual performance on child language or academic performance, mediated via household chaos (see Table S3).

**Mediation of Birth Mother Effects on Adoptive Mother Warmth via Child Language.** None of the indirect effects of birth mother intellectual performance on household chaos, via child language, were statistically significant (see Table S3).

**Direct and Indirect Effects of Birth Father Intellectual Performance on Household Chaos and Children’s Language and Academic Performance**

When birth father intellectual performance was used as a proxy for genetic influence, instead of birth mother intellectual performance, the model would not converge.

**Figure S1**

*Longitudinal Structural Equation Model Examining the Effects of Birth Mother Intellectual Performance on, and Cross-lagged Associations Between, Household Chaos and Children’s Language and Academic Performance*

*
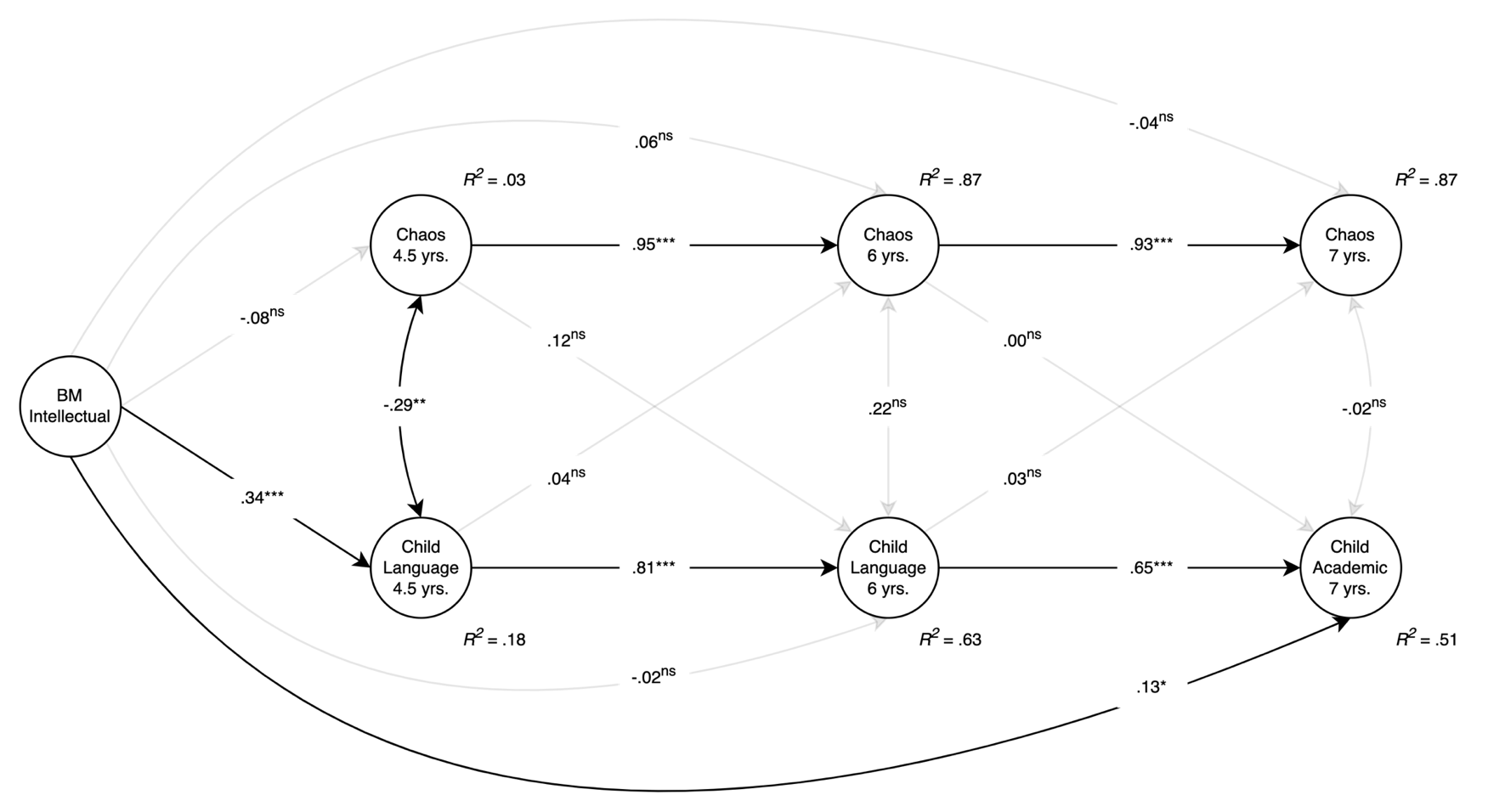
*

*Note.* Model fit: χ^2^(264) = 556, *p* < .001, CFI = .91, RMSEA = .05, SRMR = .06. Standardized estimates reported. Adoption openness, child sex, and obstetric risk were included as covariates in the model. BM = birth mother. ^***^*p* < .001. ^**^*p* < .01. ^*^*p* < .05. ^ns^*p* ≥ .1.

**Table S3**

*Direct and Indirect Effects from the Structural Equation Model Displayed in Figure S1*

| Test | Predictor | Outcome | Mediator/s | Paths | β | *p* |
| --- | --- | --- | --- | --- | --- | --- |
| H1 | BM Intellectual | Chaos 4.5 yrs. | N/A | *a* | -0.08 [-0.25, 0.10] | .401 |
| H1 | BM Intellectual | Chaos 6 yrs. | N/A | *b* | 0.06 [-0.09, 0.21] | .426 |
| H1 | BM Intellectual | Chaos 6 yrs. | Chaos 4.5 yrs. | *ag* | -0.07 [-0.24, 0.10] | .404 |
| H1 | BM Intellectual | Chaos 6 yrs. | Chaos 4.5 yrs., Child language 4.5 yrs. | *b* + *ag* + *dm*) | 0.00 [-0.17, 0.17] | .968 |
| H1 | BM Intellectual | Chaos 7 yrs. | N/A | *c* | -0.04 [-0.18, 0.10] | .567 |
| H1 | BM Intellectual | Chaos 7 yrs. | Chaos 4.5 yrs. Chaos 6 yrs. | *agh* | -0.07 [-0.23, 0.09] | .403 |
| H1 | BM Intellectual | Chaos 7 yrs. | Chaos 6 yrs. | *bh* | 0.06 [-0.08, 0.19] | .427 |
| H1 | BM Intellectual | Chaos 7 yrs. | Chaos 4.5 yrs., Chaos 6 yrs., Child language 4.5 yrs., Child language 6 yrs. | *c* + *agh* + *akn* + *bh* + *dmh* + *din* + *en* | -0.03 [-0.20, 0.14] | .745 |
| H2 | BM Intellectual | Child language 6 yrs. | Chaos 4.5 yrs, | *ak* | -0.01 [-0.03, 0.02] | .476 |
| H2 | BM Intellectual | Child academic 7 yrs. | Chaos 4.5 yrs., Child language 6 yrs. | *akj* | -0.01 [-0.02, 0.01] | .477 |
| H2 | BM Intellectual | Child academic 7 yrs. | Chaos 6 yrs. | *bl* | 0.00 [-0.01, 0.01] | .941 |
| H2 | BM Intellectual | Child academic 7 yrs. | Chaos 4.5 yrs., Chaos 6 yrs. | *agl* | 0.00 [-0.01, 0.01] | .941 |
| H2 | BM Intellectual | Child academic 7 yrs. | Chaos 4.5 yrs., Chaos 6 yrs., Child language 4.5 yrs. | (*b* + *ag* + *dm*)*l* | 0.00 [0.00, 0.00] | .971 |
| H3 | BM Intellectual | Chaos 6 yrs. | Child language 4.5 yrs. | *dm* | 0.02 [-0.05, 0.08] | .634 |
| H3 | BM Intellectual | Chaos 7 yrs. | Child language 4.5 yrs., Chaos 6 yrs. | *dmh* | 0.02 [-0.05, 0.08] | .634 |
| H3 | BM Intellectual | Chaos 7 yrs. | Child language 6 yrs. | *en* | 0.00 [-0.01, 0.01] | .816 |
| H3 | BM Intellectual | Chaos 7 yrs. | Child language 4.5 yrs., Child language 6yrs. | *din* | 0.01 [-0.03, 0.05] | .693 |
| H3 | BM Intellectual | Chaos 7 yrs. | Child language 4.5 yrs., Child language 6 yrs., Chaos 4.5 yrs. | (*e* + *di* + *ak*)*n* | 0.01 [-0.03, 0.05] | .693 |

*Note.* H1 = hypothesis 1. H2 = hypothesis 2. H3 = hypothesis 3. BM = birth mother. Values in square brackets indicate the 95% confidence intervals for the beta coefficients.

**Direct and Indirect Effects of Birth Mother Intellectual Performance on Household and Child Screen Media Use and Children’s Language and Academic Performance**

**Evocative Effects of Birth Mother Intellectual Performance on Screen Media Use.** As displayed in Figure S2 and Table S4, none of the direct, indirect, or total effects of birth mother intellectual performance (a proxy for genetic influences) on screen media use (a composite of adoptive mother and adoptive father ratings of household and child TV watching and video games) were statistically significant. The model accounted for 9% of the variance in screen media use at 4.5 years, 67% of the variance in screen media use at 6 years, and 86% of the variance in screen media use at 7 years. The large increase in the R^2^ accounted for by the model at ages 6 and 7 is primarily due to the high stability of screen use over time.

**Mediation of Birth Mother Effects on Child Language and Academic Performance via Screen Media Use.** As shown in Figure S2, the direct effect of screen media use at 4.5 years on child language at 6 years old was in the unexpected (positive) direction and statistically significant (β = 0.17, 95% CI [0.04, 0.30], *p* = .008). The direct effect of child language on screen media use at 6 years was in the expected (negative) direction and statistically significant (β = −0.14, 95% CI [−0.26, −0.01], *p* = .035). None of the other cross-lagged associations were statistically significant (see Figure S2), nor were the indirect effects of birth mother intellectual performance on child language and academic performance, via screen media use (see Table S4).

**Mediation of Birth Mother Effects on Screen Media Use via Child Language.** None of the indirect effects of birth mother intellectual performance on household and child screen media use, via child language, were statistically significant (see Table S4).

**Direct and Indirect Effects of Birth Father Intellectual Performance on Household and Child Screen Media Use and Children’s Language and Academic Performance**

When birth father intellectual performance was used as a proxy for genetic influence, instead of birth mother intellectual performance, the model would not converge.

**Figure S2**

*Longitudinal Structural Equation Model Examining the Effects of Birth Mother Intellectual Performance on, and Cross-lagged Associations Between, Household and Child Screen Media Use and Children’s Language and Academic Performance*

*
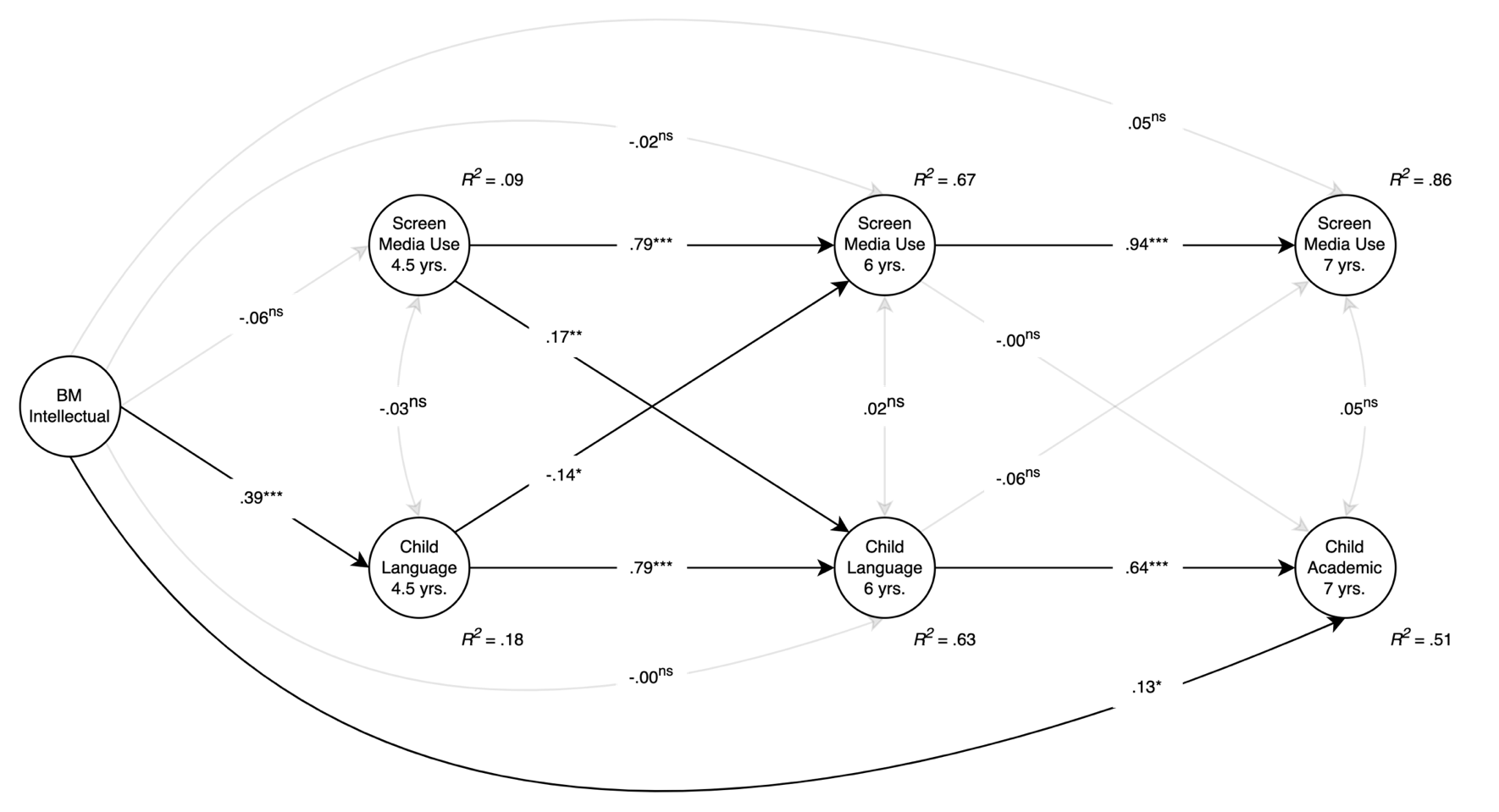
*

*Note.* Model fit: χ^2^(429) = 1305, *p* < .001, CFI = .83, RMSEA = .07, SRMR = .08. Standardized estimates reported. Adoption openness, child sex, and obstetric risk were included as covariates in the model. BM = birth mother. ^***^*p* < .001. ^*^*p* < .05. ^†^ *p* < .1. ^ns^*p* ≥ .1.

**Table S4**

*Direct and Indirect Effects from the Structural Equation Model Displayed in Figure S2*

| Test | Predictor | Outcome | Mediator/s | Paths | β | *p* |
| --- | --- | --- | --- | --- | --- | --- |
| H1 | BM Intellectual | Screen Use 4.5 yrs. | N/A | *a* | -0.06 [-0.20, 0.07] | .372 |
| H1 | BM Intellectual | Screen Use 6 yrs. | N/A | *b* | -0.02 [-0.15, 0.11] | .797 |
| H1 | BM Intellectual | Screen Use 6 yrs. | Screen Use 4.5 yrs. | *ag* | -0.05 [-0.16, 0.06] | .371 |
| H1 | BM Intellectual | Screen Use 6 yrs. | Screen Use 4.5 yrs., Child language 4.5 yrs. | *b* + *ag* + *dm*) | -0.12 [-0.26, 0.03] | .118 |
| H1 | BM Intellectual | Screen Use 7 yrs. | N/A | *c* | 0.05 [-0.05, 0.16] | .333 |
| H1 | BM Intellectual | Screen Use 7 yrs. | Screen Use 4.5 yrs. Screen Use 6 yrs. | *agh* | -0.05 [-0.15, 0.06] | .372 |
| H1 | BM Intellectual | Screen Use 7 yrs. | Screen Use 6 yrs. | *bh* | -0.02 [-0.14, 0.11] | .798 |
| H1 | BM Intellectual | Screen Use 7 yrs. | Screen Use 4.5 yrs., Screen Use 6 yrs., Child language 4.5 yrs., Child language 6 yrs. | *c* + *agh* + *akn* + *bh* + *dmh* + *din* + *en* | -0.08 [-0.22, 0.07] | .300 |
| H2 | BM Intellectual | Child language 6 yrs. | Screen Use 4.5 yrs, | *ak* | -0.01 [-0.04, 0.01] | .396 |
| H2 | BM Intellectual | Child academic 7 yrs. | Screen Use 4.5 yrs., Child language 6 yrs. | *akj* | -0.01 [-0.02, 0.01] | .397 |
| H2 | BM Intellectual | Child academic 7 yrs. | Screen Use 6 yrs. | *bl* | 0.00 [0.00. 0.00] | .969 |
| H2 | BM Intellectual | Child academic 7 yrs. | Screen Use 4.5 yrs., Screen Use 6 yrs. | *agl* | 0.00 [-0.01, 0.01] | .969 |
| H2 | BM Intellectual | Child academic 7 yrs. | Screen Use 4.5 yrs., Screen Use 6 yrs., Child language 4.5 yrs. | (*b* + *ag* + *dm*)*l* | 0.00 [-0.01, 0.01] | .969 |
| H3 | BM Intellectual | Screen Use 6 yrs. | Child language 4.5 yrs. | *dm* | -0.05 [-0.10, 0.00] | .052 |
| H3 | BM Intellectual | Screen Use 7 yrs. | Child language 4.5 yrs., Screen Use 6 yrs. | *dmh* | -0.05 [-0.10, 0.00] | .051 |
| H3 | BM Intellectual | Screen Use 7 yrs. | Child language 6 yrs. | *en* | 0.00 [-0.01, 0.01] | .944 |
| H3 | BM Intellectual | Screen Use 7 yrs. | Child language 4.5 yrs., Child language 6yrs. | *din* | -0.02 [-0.05, 0.01] | .207 |
| H3 | BM Intellectual | Screen Use 7 yrs. | Child language 4.5 yrs., Child language 6 yrs., Screen Use 4.5 yrs. | (*e* + *di* + *ak*)*n* | -0.02 [-0.05, 0.01] | .211 |

*Note.* H1 = hypothesis 1. H2 = hypothesis 2. H3 = hypothesis 3. BM = birth mother. Values in square brackets indicate the 95% confidence intervals for the beta coefficients.

**Adoption Openness Sensitivity Analysis of Exploratory Models**

We conducted a sensitivity analysis, in which the interaction term (birth parent intellectual performance * adoption openness) was included in models on warmth, household chaos, and screen media use. A summary of these findings is reported below, and the full results are available from the authors on request.

***Parenting Warmth***

When the interaction term was included in the model on adoptive mother warmth displayed in Figure 1 (main manuscript), the model fit and results did not substantially change. The only exception was that the effect size for the significant association between birth mother intellectual ability and adoptive mother warmth increased in size (β = 0.21, 95% CI [0.08, 0.34], *p* = .002). The coefficient for the interaction term was significantly negatively associated with adoptive mother warmth at that timepoint (β = −0.17, 95% CI [−0.28, −0.05], *p* = .004), but not at any other timepoint. These results suggest that, at this timepoint only, the effect of birth mother intellectual ability on adoptive mother warmth increases as adoption openness decreases. When the interaction term was included in the model on adoptive father warmth displayed in Figure 2 (main manuscript), the model fit and results did not notably change.

***Household Chaos and Screen Media Use***

When the interaction term was included in the model on household chaos (reported in Figure S1) the model fit and results did not substantially change. When it was included in the model on screen media use (reported in Figure S2) the model fit and results did not notably change. The coefficient for the interaction term was significantly negatively associated with screen media use at 6 years (β = −0.15, 95% CI [−0.27, −0.04], *p* = .011), but not at any other timepoint. These results suggest that, at this timepoint only, the effect of birth mother intellectual ability on screen media use depends on the level openness. The negative coefficient for the interaction term indicates that this effect becomes less positive (or even negative) as openness increases.

**References**

Austerberry, C., Fearon, P., Ronald, A., Leve, L. D., Ganiban, J. M., Natsuaki, M. N., . . . Reiss, D. (2022). Early manifestations of intellectual performance: Evidence that genetic effects on later academic test performance are mediated through verbal performance in early childhood. *Child Development, 93*(2), e188-e206. doi:10.1111/cdev.13706

Bernaards, C. A., & Jennrich, R. I. (2005). Gradient projection algorithms and software for arbitrary rotation criteria in factor analysis. *Educational and psychological measurement, 65*(5), 676-696. doi:10.1177/0013164404272507

Cattell, R. B. (1966). The scree test for the number of factors. *Multivariate behavioral research, 1*(2), 245-276.

Frick, P. J. (1991). *Alabama Parenting Questionnaire*. Unpublished rating scale: University of Alabama.

Johnson, A. D., Martin, A., Brooks-Gunn, J., & Petrill, S. A. (2008). Order in the House! Associations among Household Chaos, the Home Literacy Environment, Maternal Reading Ability, and Children's Early Reading. *Merrill Palmer Q (Wayne State Univ Press), 54*(4), 445-472. doi:10.1353/mpq.0.0009

Koran, J. (2020). Indicators per factor in confirmatory factor analysis: More is not always better. *Structural Equation Modeling: A Multidisciplinary Journal, 27*(5), 765-772. doi:10.1080/10705511.2019.1706527

Matheny, A. P., Wachs, T. D., Ludwig, J. L., & Phillips, K. (1995). Bringing order out of chaos: Psychometric characteristics of the confusion, hubbub, and order scale. *Journal of Applied Developmental Psychology, 16*(3), 429-444. doi:10.1016/0193-3973(95)90028-4

Melby, J. N., & Conger, R. D. (2001). The Iowa Family Interaction Rating Scales: Instrument summary. In P. K. Kerig & K. M. Lindahl (Eds.), *Family observational coding systems: Resources for systemic research* (pp. 33–58). Mahwah, NJ: Erlbaum.

Niklas, F., & Schneider, W. (2013). Home literacy environment and the beginning of reading and spelling. *Contemporary Educational Psychology, 31*(1), 40-50.

Revelle, W. (2022). *psych: Procedures for personality and psychological research*. Illinois, USA: Northwestern University.
